# Apoptosis mediated leishmanicidal activity of *Azadirachta indica* bioactive fractions is accompanied by Th1 immunostimulatory potential and therapeutic cure *in vivo*

**DOI:** 10.1186/s13071-015-0788-3

**Published:** 2015-03-26

**Authors:** Garima Chouhan, Mohammad Islamuddin, Muzamil Y Want, Malik Z Abdin, Hani A Ozbak, Hassan A Hemeg, Dinkar Sahal, Farhat Afrin

**Affiliations:** Department of Biotechnology, Parasite Immunology Laboratory, Faculty of Science, Jamia Hamdard (Hamdard University), New Delhi, 110062 India; Department of Biotechnology, Centre for Transgenic Plant Development, Faculty of Science, Jamia Hamdard (Hamdard University), New Delhi, 110062 India; Department of Medical Laboratories Technology, Faculty of Applied Medical Sciences, Taibah University, P.O. Box: 344, Universities Road, Medina, 30001 Saudi Arabia; Malaria Research Group, International Centre For Genetic Engineering and Biotechnology, Aruna Asaf Ali Marg, New Delhi, 110067 India

**Keywords:** Visceral Leishmaniasis, *Leishmania donovani*, Apoptosis, Immunomodulatory, Antileishmanial, Immunopotentiating, *Azadirachta indica*, Immunostimulatory, Leishmanicidal

## Abstract

**Background:**

Exploration of immunomodulatory antileishmanials of plant origin is now being strongly recommended to overcome the immune suppression evident during visceral leishmaniasis (VL) and high cost and toxicity associated with conventional chemotherapeutics. In accordance, we assessed the *in vitro* and *in vivo* antileishmanial and immunomodulatory potential of ethanolic fractions of *Azadirachta indica* leaves (ALE) and seeds (ASE).

**Methods:**

*A. indica* fractions were prepared by sequential extraction of the powdered plant parts in hexane, ethanol and water. Erythrosin B staining was employed to appraise the anti-promastigote potential of ALE and ASE. Cytostatic or cytocidal mode of action was ascertained and alterations in parasite morphology were depicted under oil immersion light microscopy. Study of apoptotic correlates was performed to deduce the mechanism of induced cell death and anti-amastigote potential was assessed in *Leishmania* parasitized RAW 264.7 macrophages. *In vivo* antileishmanial effectiveness was evaluated in *L. donovani* infected BALB/c mice, accompanied by investigation of immunomodulatory potential of ALE and ASE. Adverse toxicity of the bioactive fractions against RAW macrophages was studied by MTT assay. *In vivo* side effects on the liver and kidney functions were also determined. Plant secondary metabolites present in ALE and ASE were analysed by Gas chromatography–mass spectrometry (GC-MS).

**Results:**

ALE and ASE (500 μg ml^−1^) exhibited leishmanicidal activity in a time- and dose-dependent manner (IC_50_ 34 and 77.66 μg ml^−1^, respectively) with alterations in promastigote morphology and induction of apoptosis. ALE and ASE exerted appreciable anti-amastigote potency (IC_50_ 17.66 and 24.66 μg ml^−1^, respectively) that was coupled with profound *in vivo* therapeutic efficacy (87.76% and 85.54% protection in liver and 85.55% and 83.62% in spleen, respectively). ALE exhibited minimal toxicity with selectivity index of 26.10 whereas ASE was observed to be non-toxic. The bioactive fractions revealed no hepato- and nephro-toxicity. ALE and ASE potentiated Th1-biased cell-mediated immunity along with upregulation of INF-γ, TNF-α and IL-2 and decline in IL-4 and IL-10 levels. GC-MS analysis revealed several compounds that may have contributed to the observed antileishmanial effect.

**Conclusion:**

Dual antileishmanial and immunostimulatory efficacy exhibited by the bioactive fractions merits their use alone or as adjunct therapy for VL.

**Electronic supplementary material:**

The online version of this article (doi:10.1186/s13071-015-0788-3) contains supplementary material, which is available to authorized users.

## Background

Leishmaniasis is a complex poverty associated vector-borne illness that presents a distinct array of clinical patterns and includes visceral leishmaniasis (VL), which is potentially fatal, if left untreated. VL, also known as Kala-azar is attributable to parasites of *L. donovani* complex and is characterized by fever (usually with rigor and chills), cachexia, anaemia, hepato- and spleno-megaly, pancytopenia, polyclonal hypergammaglobulinemia and hyperpigmentation resulting in characteristic darkening of skin. More than 90% of disease burden (60,000 deaths and incidence of 500,000 cases per annum) is borne by India, Bangladesh, Nepal, Sudan, Ethopia, and Brazil. Around 200 million people living in 109 districts in India, Bangladesh and Nepal are at constant risk of developing visceral infection and relapse after complete cure in the form of post kala-azar dermal leishmaniasis further adds to the complications. The escalating drug resistance exhibited by the *Leishmania* parasites had already led to withdrawal of the first line drugs from therapeutic regimen in India. By evolving as a co-infection with HIV, VL has expanded its niche to other non-endemic countries, and has managed to seek immediate attention towards its timely control [1,2]. In case of parasitic diseases, recovery is often associated with direct parasite killing in synergy with activation of the depressed immune system [3]. Progressive VL is also characterized by profound suppression of the immune system, which results in acute parasitic load. Thus, drugs that can simultaneously cause parasite death as well as activation of cell mediated immunity (CMI) can surely benefit the crippled chemotherapy against leishmaniasis. Use of medicinal plants as antidotes against parasitic infections is well-documented and herbal preparations that possess dual anti-parasitic and immunomodulatory properties have been pivotal to many classical systems of medicine. A huge number of plants with immunomodulatory potential have been explored for antileishmanial activity and have yielded promising results [4,5].

*Azadirachta indica* (neem) belonging to the family Meliaceae has become a focus of modern medicine as it has been used since ages in Ayurveda, Unani and Homeopathic systems of medicine. Every part of the neem tree possesses a wide array of biologically active compounds with medicinal potency. *A. indica* oil and the bark and leaf extracts have therapeutic efficacy against leprosy, intestinal helminthiasis and respiratory disorders in children. Leaf and seed extracts possess immunomodulatory, insecticidal, antiseptic, antitumor, anticancer, antiviral, antifungal and antiprotozoal properties ([6] and references therein). Chloroform extracts of *A. indica* leaves distinctly restrained growth of *Trypanosoma cruzi* epimastigotes with ultrastructural alterations indicated by vacuolization, organelle disintegration and disruptions in cell division [7]. *A. indica* extracts have also been demonstrated to be antileishmanial against new world species both, *in vitro* [8-10] and *in vivo* [11]. Aqueous extracts of *A. indica* whole plant [12] and of different parts [13] have been earlier reported to be leishmanicidal *in vivo* and *in vitro* against *L. donovani* promastigotes (Dd8 and other strains) but none of the studies elucidated the detailed mechanism of action of these extracts. Thus, these studies provoked us to formulate different fractions of *A. indica,* evaluate their antileishmanial potential and illustrate the mechanism of induced cell death as well as to identify the plant secondary metabolites, which could be responsible for the observed leishmanicidal potency.

## Methods

M199 medium, Roswell Park Memorial Institute (RPMI) 1640 medium, penicillin G sodium salt, streptomycin sulphate, propidium iodide (PI), glutamine and pentamidine were purchased from Sigma-Aldrich, fetal bovine serum (FBS) from Biowest, dimethylsulfoxide (DMSO) from SRL, 4-(2-hydroxyethyl)-1-piperazine-ethanesulfonic acid (HEPES) from Himedia, isopropanol and methanol from Merck. Annexin V–FLUOS staining kit and ApoDirect kits were procured from Roche Inc., Switzerland. All the antibodies and their isotype controls were procured from BD Biosciences. All the other chemicals were obtained from Sigma-Aldrich unless stated otherwise.

### Parasite culture and maintenance

*L. donovani* infection (MHOM/IN/83/AG83) was maintained by passage in BALB/c mice. Medium 199 (M199), pH 7.4 supplemented with penicillin G sodium (100 U ml^−1^), streptomycin sulfate (100 μg ml^−1^), HEPES (25 mM) and 10% heat-inactivated FBS (complete M199) was used to culture the promastigotes. Subculturing was done at regular intervals (72–96 h) after adjusting the inoculum density to 2 × 10^6^ cells ml^−1^ at 22°C [14].

### Macrophage culture and maintenance

Murine macrophage-like adherent cell line RAW 264.7 was maintained in RPMI 1640 medium in a humidified atmosphere and 5% CO_2_ at 37°C. The medium was supplemented with 100 μg ml^−1^ streptomycin sulfate, 100 U ml^−1^ penicillin G-sodium, 0.2% sodium bicarbonate, 25 mM HEPES and 10% FBS. The cell line was sub-cultured every 3 days in the same medium [15].

### Animals

For *in vivo* experiments, a prior consent was obtained from the Jamia Hamdard Animal Ethics Committee (JHAEC) for the research protocol (Ethical approval judgement number 499). JHAEC is registered under the Committee for the purpose of control and supervision of experiments on animals (CPCSEA). In present study, female BALB/c mice at 6–8 weeks of age and weighing about 20–25 g were used. All the animals were individually placed in standard size polycarbonate cages with controlled conditions of temperature (23 ± 1°C), humidity (55 ± 10%) and 12:12 h of light and dark cycles. The animals were fed with a standard pellet diet (Ashirwad Industries, Chandigarh, India) and filtered water *ad libitum* in the Central Animal House of Jamia Hamdard in accordance with internationally established principles.

### Preparation of plant fractions

*A. indica* bark, leaves and seeds were obtained from the Herbal Garden of Jamia Hamdard. The plant samples were thoroughly washed with distilled water, shade dried, ground to powder form and sequentially extracted in hexane, ethanol and water by percolation [16]. The respective fractions were pooled and concentrated to dryness under vacuum by rotary evaporation at 35°C. The dried solvent and lyophilized aqueous fractions were stored at −20°C until used for bioassay.

### Evaluation of anti-promastigote potential of *A. indica* fractions

Anti-promastigote activity was appraised by incubating *L. donovani* promastigotes with different *A. indica* fractions followed by enumeration of the viable cells after erythrosin B (0.2%, 1:1) staining. Briefly, stationary phase promastigotes (2 × 10^6^ cells ml^−1^) were incubated at 22°C with or without the test fractions (500 μg ml^−1^) or pentamidine (a known antileishmanial drug, serving as positive control) at equivalent concentration. DMSO (0.5%), which was used to solubilize the *A. indica* fractions, was also assayed in parallel with the promastigotes to determine any unspecific parasite death and served as solvent control. The viable cells that excluded the dye erythrosin B were then counted at 24 h interval for 7 days under phase contrast microscope (40 X) in a hemocytometer [16].

### Growth reversibility assay

The bioactive fractions of *A. indica* from the growth kinetics assay were examined for their cytostatic or cytocidal potential. Thus, only *A. indica* leaves ethanolic (ALE) and *A. indica* seeds ethanolic (ASE) fractions were included in this assay. In brief, the treated or untreated parasites were harvested (7 days post incubation with the bioactive fractions), washed twice (3000 × g, 10 min, 4°C) with incomplete M199 (without FBS) and finally resuspended in fresh complete M199 at 22°C. After 96 h, the cell viability was ascertained by counting the live promastigotes under the microscope, which were distinguished from dead cells by erythrosin B staining.

### Assessment of 50% promastigote growth inhibitory concentration (IC_50_) of the bioactive fractions

Stationary phase promastigotes (2 × 10^6^ ml^−1^) were incubated with the bioactive fractions or pentamidine or medium alone at serial three fold dilutions (500 to 2.05 μg ml^−1^). After 96 h, parasite survival was assessed by enumerating the live cells. Percent (%) viability was determined in line with the formula:

$$ \%\ \mathrm{Viability} = \frac{\mathrm{Average}\ \mathrm{viable}\ \mathrm{cell}\ \mathrm{count}\ \mathrm{per}\ {\mathrm{ml}}_{\left(\mathrm{treated}\ \mathrm{samples}\right)}\ }{{\mathrm{Average}\ \mathrm{viable}\ \mathrm{cell}\ \mathrm{count}\ \mathrm{per}\ \mathrm{ml}}_{\left(\mathrm{parasite}\ \mathrm{control}\right)}} \times 100 $$

IC_50_, the concentration that reduced parasite growth by 50% was evaluated by graphical extrapolation.

### Study of cellular morphology

Morphological alterations in *Leishmania* parasites post-treatment with ALE and ASE were studied. In brief, the *L. donovani* promastigotes (2 × 10^6^ ml^−1^) were cultured without or with ALE, ASE or pentamidine (500 μg ml^−1^) for 96 h, following which the parasites were harvested (3000 × g, 10 min, 4°C) and washed twice with phosphate buffered saline (PBS, 0.02 M, pH 7.4). The parasites from all experimental groups were fixed with 80% ethanol, stained with erythrosin B at room temperature and the samples observed under Nikon Eclipse 80i microscope (100 X objective) and the photomicrographs taken. Here, erythrosin B was employed to stain the cells for optimal imaging rather than to distinguish live and dead cells.

### Phosphatidylserine (PS) exposure in bioactive fractions-treated promastigotes

To investigate the apoptotic mode of cell death, PS externalization by means of Annexin –V FLUOS binding was studied. The assay is based upon the ability of Annexin-V-conjugated with a flurochrome to bind with PS, which flips over to the cell surface during apoptosis. To further differentiate between apoptotic and necrotic cells, PI was added as it penetrates the cells with compromised membrane integrity (dead cells or late apoptotic cells). Briefly, promastigotes (2 × 10^6^ ml^−1^) were cultured with the bioactive fractions or pentamidine (500 μg ml^−1^) for 72 h. The treated and untreated cells were harvested (3000 × g, 10 min, 4°C) and washed twice with PBS. Annexin-V FLOUS and PI staining was performed using Annexin –V FLUOS staining kit (Roche) according to the manufacturer’s instructions. The samples were acquired on a BD LSR-II flow cytometer and the dot plots of FLUOS fluorescence versus PI were recorded. Subsequently, data were analysed using BD FACS DIVA software.

### Detection of DNA fragmentation in bioactive fractions treated promastigotes by terminal deoxynucleotidyltransferase (TdT) mediated dUTP (deoxyuridine triphosphate) nick end labeling (TUNEL) assay

Internucleosomal DNA fragmentation is an index for apoptosis or programmed cell death (PCD) which can be detected by TUNEL staining. This assay employs the use of enzymatic activity of TdT, which incorporates labeled dUTP into free 3′-OH (hydroxyl) termini of the fragmented DNA. Briefly, parasites (2 × 10^6^ ml^−1^) with or without treatment (500 μg ml^−1^, 72 h) were harvested (3000 × g, 10 min, 4°C), washed with PBS and fixed with 4% paraformaldehyde for 1 h on ice. The downstream processing was carried out according to the manufacturer’s instructions as described elsewhere [16]. Acquisition of cells was performed on a BD FACS LSR-II flow cytometer. Histograms were recorded for each sample and the shift in mean fluorescence intensity (MFI) along x-axis (FL-1H, FLOUS fluorescence) in treated cells was compared with that of untreated labeled promastigotes.

### Analysis of cell cycle distributions by propidium iodide (PI) staining

Cell cycle analysis reveals distribution of cells in three interphase stages of cell cycle, *i.e.*, G_0_/G_1_, S, and G_2_/M and also allows the detection of apoptotic cells with fractional DNA content which was quantified by staining with PI according to Sarkar et al., [17] with few modifications. In brief, the promastigotes were either cultured in the presence or absence of bioactive fractions (500 μg ml^−1^) along with appropriate controls for 72 h. The promastigotes were harvested (3000 × g, 10 min, 4°C), washed twice with PBS followed by fixation with 80% chilled ethanol and kept at 4°C at least for 24 h. The fixed cells were harvested, washed twice with PBS, and incubated with RNase (200 μg ml^−1^) for 1 h at 37°C and stained with PI (50 μg ml^−1^) for 20 min in dark at 22–25°C. The cells were acquired using a BD LSR II flow cytometer and cytofluorimetric analysis of cell cycle distributions was done using BD FACS DIVA software.

### Determination of Mitochondrial Membrane Potential

Alterations in mitochondrial membrane potential (Ψm) were depicted flow cytometrically using a cell permeable dye JC-1 (5,59,6,69-tetrachloro-1,19,3,39-tetraethylbenzimidazolylcarbo-cyanineiodide). In live normal cells, JC-1 accumulates and aggregates in mitochondria and emits red fluorescence at 590 nm. Whereas, in the cells undergoing apoptosis, where there is disruption of mitochondrial membrane; JC-1 exists in cytoplasm as monomers, emitting green fluorescence (530 nm). Thus, the fluorescence intensity ratio 590/530 nm typically represents relative Ψm of a cell. Briefly, to assess the changes in Ψm, *L. donovani* promastigotes (2 × 10^6^ ml^−1^) after treatment with ALE, ASE and pentamidine (500 μg ml^−1^), were harvested (3000 × g, 10 min, 4°C), washed twice with PBS and stained with JC-1 (10 μg, 10 min, 37°C). The cells were again washed twice with PBS followed by acquisition in a BD LSR II flow cytometer [18].

### Detection of intracellular reactive oxygen species (ROS)

Intracellular ROS generation induced by the bioactive fractions was monitored by H_2_DCFDA (2′, 7′- dichlorodihydrofluorescein-diacetate) staining. *L. donovani* promastigotes (2 × 10^6^ ml^−1^) were incubated without or with the bioactive fractions (500 μg ml^−1^) at 22°C for 72 h. Cells were washed twice with PBS, and incubated with H_2_DCFDA (10 μM) at room temperature in dark. After 15 min, the intensity of fluorescent signal of each sample was acquired in a BD LSR II flow cytometer and the MFI was recorded and represented in the form of respective histograms.

### Assessment of anti-amastigote activity *ex vivo*

To determine the antileishmanial efficacy of *A. indica* fractions, macrophage-like cell line RAW 264.7 was parasitized with *L. donovani* promastigotes and treated with ALE or ASE. In brief, RAW macrophages (5 × 10^6^ ml^−1^, 100 μl per well in RPMI-1640 medium) were plated on round cover slips (13 mm diameter, Himedia) that were placed in 24-well plates (Corning) for 24 h in a carbon dioxide incubator (5% CO_2_) at 37°C. The coverslips were washed thrice with serum free RPMI 1640 to remove the non-adherent cells. The cells were infected with promastigotes in stationary-phase at a ratio of 1: 10 (macrophage: *Leishmania*) for 24 h. Cellular monolayers were washed to remove the non-phagocytosed promastigotes. Infected macrophages were either incubated in media alone (infection control) or with serial four-fold dilutions of ALE, ASE or pentamidine to achieve final concentration between 200 to 3.12 μg ml^−1^. After 48 h, the cover slips were washed with PBS, dried, fixed with chilled methanol, giemsa-stained and examined under light microscope. A minimum of 200 macrophages were counted per cover slip to determine the number of resident amastigotes. Percent reduction was determined using the formula:

$$ \%\ \mathrm{Reduction} = \frac{\mathrm{Number}\ \mathrm{of}\ \mathrm{amastigotes}\ \mathrm{per}\ 200\ {\mathrm{macrophages}}_{\left(\mathrm{treated}\ \mathrm{samples}\right)}\ }{{\mathrm{Number}\ \mathrm{of}\ \mathrm{amastigotes}\ \mathrm{per}\ 200\ \mathrm{macrophages}}_{\left(\mathrm{infected}\ \mathrm{control}\right)}} \times 100 $$

IC_50_, the drug concentration that is cytotoxic to 50% of the amastigotes, was calculated by plotting the percent reduction in amastigotes against drug concentrations tested [19].

### Cytotoxic potential of the bioactive fractions and determination of selectivity index (SI)

The cytotoxicity of ALE and ASE against mammalian macrophages was measured by MTT [3-(4,5-dimethylthiazol-2-yl)-2,5-diphenyl tetrazolium bromide] assay on RAW 264.7 cells. Briefly, macrophages (2 × 10^6^ ml^−1^) in RPMI 1640 were seeded in 96 well tissue culture plates (200 μl well^−1^) for 24 h in a CO_2_ incubator (5% CO_2_, 37°C). The non-adherent cells were washed away with incomplete media and the adherent macrophages were exposed to different dilutions of the bioactive fractions (0 to 500 μg ml^−1^) at serial two fold dilutions in triplicates for 48 h. MTT (5 mg ml^−1^, 50 μl well^−1^) was added for further incubation of 4–6 h in a CO_2_ incubator. Following incubation, the plate was centrifuged (400 × g, 20 min, 4°C) and the supernatant was aspirated. The resulting formazan precipitate was dissolved in isopropanol: dimethylsulfoxide (1:1). The relative amount of formazan produced by the viable cells was quantified spectrophotometrically at 570 nm in an ELISA plate reader. The absorbance of untreated macrophages was considered as 100%. Percent viability for different experimental groups was calculated as:

$$ \%\ \mathrm{Viability} = \frac{\mathrm{Mean}\ \mathrm{specific}\ {\mathrm{absorbance}}_{\left(\mathrm{treated}\ \mathrm{samples}\right)}\ }{{\mathrm{Mean}\ \mathrm{specific}\ \mathrm{absorbance}}_{\left(\mathrm{control}\ \mathrm{samples}\right)}} \times 100 $$

Dose response curves were plotted and concentration of fractions that is cytotoxic to 50% macrophages (CC_50_) was obtained through graphical extrapolation. CC_50_ concentration thus obtained was used to determine SI, which is defined as specificity of the bioactive fractions, against internalized amastigotes over host macrophages. SI was calculated according to the formula:

$$ \mathrm{S}\mathrm{I} = \frac{{\mathrm{CC}}_{50}\kern0.5em \mathrm{against}\ \mathrm{macrophages}}{{\mathrm{IC}}_{50}\kern0.5em \mathrm{against}\ \mathrm{amastigotes}} $$

### GC-MS analysis of ALE and ASE

To identify the plant secondary metabolites that may have contributed to the observed antileishmanial efficacy of ALE and ASE, GC-MS analysis was performed as described previously [16] using a Shimadzu QP2010 equipped with a DB-5MS column (30 m length, 0.25 mm i.d., film thickness 0.25 μm). The column temperature was increased gradually from 60 to 310°C at 5°C min^−1^, and the injector and detector temperature was 260°C. Helium was employed as the carrier gas, set at a constant flow rate of 1.5 ml min^−1^. The mass spectrums were produced in an electron impact ionization mode of 70 eV and the chemical constituents identified after correlation of the recorded mass spectra with the reference spectra of WILEY8.LIB and NIST08.LIB library, supplied with the software of the GC-MS system.

### *In vivo* experimental plan

For infection, stationary phase *L. donovani* promastigotes were administered intravenously to 6 to 8-week old female BALB/c mice (2.5 × 10^7^/animal). Ten weeks post-infection, parasite load was verified in three randomly selected animals by microscopic examination of giemsa-stained tissue smears of liver and spleen as well as parasite transformation from splenocytes after which, the mice were arbitrarily assorted into different groups (n = 5 in each group) and the treatment was initiated on the next day according to the regime (Table 1). Treatment was carried out for 2 weeks daily at the mentioned doses except for amphotericin B (AmB, a known antileishmanial drug), which was administered alternatively over a period of 10 days at the indicated dose. Mice were bled 2 weeks post treatment and sacrificed for assessment of infection and study of different immunomodulatory and toxicity parameters.

**Table 1:** **Experimental design for** ***in vivo*** **studies**

Group 1 (Normal)	Normal control/ Naïve mice (Administered PBS only)
Group 2 (INF)	Infected control (BALB/c mice infected with *L. donovani* promastigotes and administered PBS)
Group 3 (VC)	Vehicle control (0.5% DMSO in PBS, the highest concentration used to dissolve the fractions/compounds)
Group 4 (AmB)	Amphotericin B administered intravenously (i.v.) at (5 mg/kg bw)
Group 5 (ALE100)	ALE administered intraperitoneally (i.p.) at 100 mg/kg bw
Group 6 (ALE 200)	ALE administered i.p. at 200 mg/kg bw
Group 7 (ASE100)	ASE administered i.p. at 100 mg/kg bw
Group 8 (ASE200)	ASE administered i.p. at 200 mg/kg bw

### Assessment of organ parasite burden

To assess the *in vivo* efficacy of *A. indica* bioactive fractions, hepatic and spleen parasitic load was determined in treated and untreated animals. Mice were euthanized at 2 weeks post treatment and liver and spleen weights were measured. Small pieces of tissue each from liver and spleen were incised to prepare impression smears. The tissue impressions were dried, fixed with chilled absolute methanol and subsequently giemsa-stained. The parasite burden was evaluated as Leishman-Donovan Units (LDU) according to the formula [20]:

$$ \frac{\mathrm{Number}\ \mathrm{of}\ \mathrm{amastigotes}\ }{\mathrm{Number}\ \mathrm{of}\ \mathrm{macrophages}} \times \mathrm{organ}\ \mathrm{weight}\ \left(\mathrm{mg}\right) $$

Further, percent protection rendered by the different treatment groups was calculated according to the formula:

$$ \frac{\mathrm{LD}{\mathrm{U}}_{\mathrm{infected}\ \mathrm{control}}\hbox{--}\ \mathrm{L}\mathrm{D}{\mathrm{U}}_{\mathrm{treated}}\ }{\mathrm{LD}{\mathrm{U}}_{\mathrm{infected}\ \mathrm{control}}} \times 100 $$

### Preparation of leishmanial antigens

*Leishmania* freeze thawed antigen (FT) was prepared from stationary phase promastigotes. Briefly, the promastigotes (10^8^ ml^−1^) were harvested, washed twice with PBS and subjected to 6 cycles of alternate freezing (at −70°C, 30 min) and thawing (37°C, 15 min) followed by estimation of protein concentration by Folin and Lowry method [21]. Soluble leishmanial antigen (SLA) was prepared similarly with some additional modifications. Parasites (10^8^ ml^−1^) were lysed by 8 freeze-thaw cycles as explained above, followed by centrifugation at 5250 × g for 30 min [22]. The resultant supernatant was separated and protein concentration determined. The antigens were stored at −70°C until use.

### Evaluation of delayed type hypersensitivity (DTH) responses

It is well established in leishmaniasis that successful cure results in transition from DTH-negative to DTH-positive state. Thus, DTH response which serves as a marker of CMI was measured in all the test animals two weeks post-treatment. Briefly, mice right and left hind foot pads were inoculated intradermally with FT (800 μg ml^−1^) and PBS alone, respectively [23]. Footpad swelling was measured after 24 h of antigen inoculation by a vernier calipers and the DTH response (mm) was measured as the mean difference between the footpad swelling of two hind footpads.

### Detection of serum IgG isotypes

The production of immunoglobulin G (IgG) isotypes directly relates to the induction of T-helper (Th) subsets of CD4^+^ T cells, which defines the outcome of immune response via polarization towards either cellular (Th1) or humoral immune response (Th2). Thus, to assess whether our fractions activated CMI, serum levels of IgG isotypes, IgG2a and IgG1 were assessed by means of ELISA. Briefly, mice were bled 2 weeks post treatment and the sera was separated and stored at −70°C until use. 96 well U-bottom plates (Corning) were coated with FT (25 μg ml^−1^) and left overnight at 4°C. To remove unbound antigen, the plates were gently washed (2 min × 3 times) with washing buffer (PBS with 0.05% Tween-20) and the non-specific sites were blocked by 1% BSA in PBS for 3–4 h at room temperature. After washing, the plates were incubated with mice sera (1: 1000, diluted in PBS) overnight followed by washing to remove unbound primary antibodies. The plates were then incubated with secondary antibodies, goat anti-mouse IgG2a and IgG1 (Sigma) followed by washing and further incubation with tertiary antibody, *i.e.*, rabbit anti-goat IgG conjugated with horse radish peroxidase (1: 10,000). The plates were again washed with washing buffer thrice and finally with PBS followed by addition of the substrate solution (o-phenylenediamine dihydrochloride, 0.8 mg ml^−1^ in phosphate citrate buffer, pH 5.0 with 0.04% H_2_O_2_). The plates were incubated for 5 min and read at 450 nm in an ELISA reader [24].

### Quantification of nitric oxide (NO)

Th1 cells activate nitric oxide synthase II (NOS II) mediated conversion of L-arginine into NO and citrulline. NO is a potent cytotoxic molecule majorly responsible for containing intracellular parasites including *Leishmania.* In cell culture, NO is detected in the form of nitrite which is one of the two primary, stable and non-volatile products of NO. Briefly, splenocytes from all experimental groups, unstimulated or primed with SLA (12 μg ml^−1^) were incubated for 48 h as described above. The culture supernatants were harvested, to which an equal volume of Griess reagent [1% sulphanilamide and 0.1% N-(1-naphthyl) ethylenediamine dihydrochloride in 5% H_3_PO_4_] was added and the plates incubated for 10 min at room temperature followed by absorbance measurement in ELISA plate reader at 540 nm. The nitrite levels in the different experimental groups were quantified with respect to the standard curve generated with sodium nitrite.

### Determination of lymphoproliferative response

To study the *in vitro* recall response, lymphoproliferation as an index of CMI was evaluated in spleen cells by labeling with carboxyfluorescein succinimidyl ester (CFSE). Briefly, animals from all the experimental groups were euthanized 2 weeks post-treatment and the spleens were aseptically removed. Single cell suspension of the spleen was prepared in RPMI-1640 incomplete media and filtered through 0.22 μm nylon mesh (BD Biosciences). The red blood cells (RBCs) were lysed with ice-cold lysis buffer (20 mM Tris buffered with 0.14 M NH_4_Cl). After several washes with incomplete RPMI medium (without FBS), the viable cells were counted after trypan blue dye exclusion. CFSE staining procedure was followed according to Del-Rey et al., [25] with few modifications. The splenocytes at a cell density of 5 × 10^6^ cells ml^−1^ were incubated with 1 μM of CFSE, in dark at room temperature for 15 min, and the reaction was quenched with ice cold complete RPMI-1640 media (with 10% FBS). The cells were washed twice with the same medium and seeded in 96 well flat bottom plates (Corning). The cells were re-stimulated *in vitro* either with medium alone or with SLA (12 μg ml^−1^) or with Concanavalin A (ConA, 5 μg ml^−1^), a non-specific mitogen inducer, taken as positive control. After 48 h, the cells were washed twice with PBS and finally resuspended in PBS. 50,000 cellular events were acquired for each sample in a flow cytometer, histograms were plotted and the percentage of cells undergoing proliferation was deduced from the histogram statistics.

### Study of CD4 and CD8 T cell expression profile

Since CD4 as well as CD8 T cells are essential to successful cure of leishmaniasis, CD4 and CD8 T cell populations were characterized in splenocytes by surface phenotyping. Briefly, 2 weeks post treatment, the spleens were homogenized to single cell suspension in RPMI 1640 medium and the RBCs lysed as described above. After sufficient washings, the live cells excluding trypan blue were enumerated. A total of 2 × 10^6^ cells per tube were taken for downstream processing. The cells were washed twice with FACS buffer (PBS with 1% BSA), and subsequently stained with antimouse-CD4-phycoerythrin (PE) and antimouse-CD8-fluorescein isothiocyanate (FITC) antibodies diluted (1: 200) in the same buffer for 15 min in dark on ice. Following incubation, the cells were washed twice and finally acquired in a BD LSR II flow cytometer. Suitable isotype controls were also included to rule out background fluorescence. Dot plots were generated and quadrants were set, quadrant statistics revealed the percentage of each positive population [26].

### Immunostimulatory effect of ALE and ASE on macrophages

Co-stimulatory molecules present on antigen presenting cells (APCs) play a pivotal role in outcome of leishmaniasis since T cell-mediated activation of APCs is largely dependent on proper functioning of these molecules owing to their contribution in T cell receptor-major histocompatibility complex (MHC) interactions. Thus, the expression of CD80 and CD86 co-stimulatory molecules was studied in peritoneal cavity derived macrophages, 2 weeks post-treatment. Animals from different experimental groups were sacrificed and macrophages were drawn from peritoneal cavity aseptically in incomplete RPMI-1640 medium. The cells were washed twice with incomplete medium and 2 × 10^6^ macrophages per tube were taken and washed with FACS buffer. The same buffer was used for all subsequent washings and to prepare antibody dilutions. Following two washes, the cells were co-stained with Allophycocyanin (APC) conjugated antimouse-CD80, antimouse-CD86-PeCy7 (a tandem fluorochrome) (1: 200 dilution). Single flurochrome controls and isotype controls were accordingly prepared to assess the staining intensity and background staining as well as to adjust the compensation. Staining procedure was carried out in dark for 15 min on ice followed by two subsequent washes and acquisition of cells in a BD LSR II flow cytometer.

### Detection of Th1/Th2 cytokines

Shift in pro-inflammatory and anti-inflammatory cytokine secretion patterns can directly determine the outcome of *Leishmania* infection. Hence, the Th1/Th2 cytokines levels were determined in culture supernatant of splenic lymphocytes. Briefly, lymphocytes from spleens of untreated and treated animals were pulsed *in vitro* with 12 μg ml^−1^ of SLA in a humidified atmosphere at 37°C. Post 48 h, the culture supernatant from all the experimental groups was harvested and stored at −70°C until analysis for INF-γ, TNF-α, IL-2, IL-4 and IL-10 using mouse flex set, BD cytometric bead array in accordance with the manufacturer’s instructions [23].

### Estimation of *in vivo* toxicity of ALE and ASE on liver and kidney functions

To assess the *in vivo* toxicity of bioactive fractions, naïve mice (5 per group) were injected with ALE, ASE (i.p) and AmB (i.v) as per earlier described regimen. The mice were dosed continuously for 14 days and 2 weeks post-treatment, the mice were bled to obtain serum samples. To evaluate the side effects on liver function, serum glutamate pyruvate transaminase (SGPT), serum oxaloacetate transaminase (SGOT) and alkaline phosphatase (ALP) were measured using commercially available kits (Span Diagnostics Ltd., Surat, India). To determine the adverse effects of the bioactive fractions on kidney function, urea and creatinine concentrations were estimated in the respective serum samples by commercially available kits (Span Diagnostics Ltd., Surat, India) according to the manufacturer’s instructions.

### Statistical analysis

All *in vitro* experiments were replicated at least thrice in triplicate and the results depicted are from one of the three independent experiments and expressed as mean ± standard error of mean (SEM) of samples. The *in vivo* study was carried out twice with 5 mice per group, and the data are from one of the two independent experiments and expressed as mean ± SEM. Statistical analysis was performed using Graph-Pad Prism 5 software and the statistical significance was calculated by one-way analysis of variance (ANOVA) followed by Tukey’s multiple comparison test. Differences were considered statistically significant at *P* < 0.05.

## Results

### ALE and ASE inhibit the growth of *L. donovani* promastigotes

**Figure 1:**
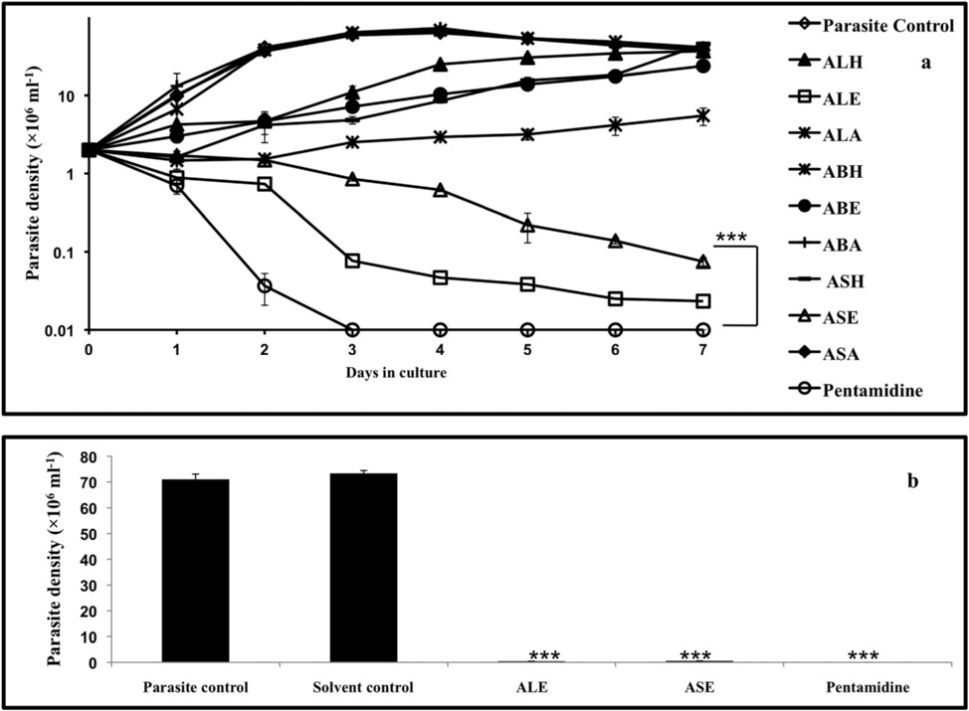
**Antileishmanial effect of** ***A. indica*** **fractions on** ***L. donovani*** **promastigotes. (a)**. Growth Kinetics Assay. 2 × 10^6^ ml^−1^ stationary phase promastigotes were grown in the presence of different *A. indica* fractions: *A. indica* leaves hexane (ALH), ethanol (ALE) and aqueous (ALA); *A. indica* bark hexane (ABH), ethanol (ABE) and aqueous (ABA); *A. indica* seeds hexane (ASH), ethanol (ASE) and aqueous (ASA). Additional experimental groups included parasite control (without any treatment), and pentamidine. ***P < 0.001 in comparison to parasite control. **(b)**. Growth Reversibility Analysis. *L. donovani* promastigotes (2 × 10^6^ ml^−1^) were incubated with ALE, ASE, pentamidine and DMSO (solvent control, 0.5%) and growth reversal after drug treatment was analyzed as described in Methods. ***P < 0.001 with respect to parasite control.

The growth inhibitory effect of *A. indica* leaves, bark and seed fractions was assessed against exponentially growing *L. donovani* promastigotes. ALE and ASE reduced the proliferation of *L. donovani* promastigotes in a time-dependent manner with ALE being more effective in reducing the parasite growth than ASE (Figure 1a). *A. indica* bark hexane fraction (ABH) initially suppressed parasite multiplication, but a gradual increase in cell density was evident. None of the other fractions caused significant decline in parasite viability. Pentamidine, an established antileishmanial compound rapidly eliminated *Leishmania* parasites *in vitro*. Parasites without any treatment or with 0.5% DMSO (data not shown) proliferated at normal rates confirming the inertness of the solvent.

### ALE and ASE induced mode of parasite killing is leishmanicidal

To determine whether the mode of ALE and ASE induced parasite death is leishmanistatic or leishmanicidal, the parasites from different experimental groups (after incubation for 7 days) were washed to remove the test fractions/compounds and were further incubated for 96 h in fresh complete medium. Marginal number of parasites reverted back to growth in both ALE (0.43 × 10^6^ ml^−1^ ± 0.021, *P* < 0.001) and ASE (0.57 × 10^6^ ml^−1^ ± 0.013, *P* < 0.001) treated samples, in comparison to control without treatment and as well as vehicle control (treated with 0.5% DMSO), indicating that the mode of cell death is predominantly cytocidal in nature. In pentamidine treated samples, no reversion in parasite growth was observed (Figure 1b).

### IC_50_ of bioactive fractions on promastigotes

**Figure 2:**
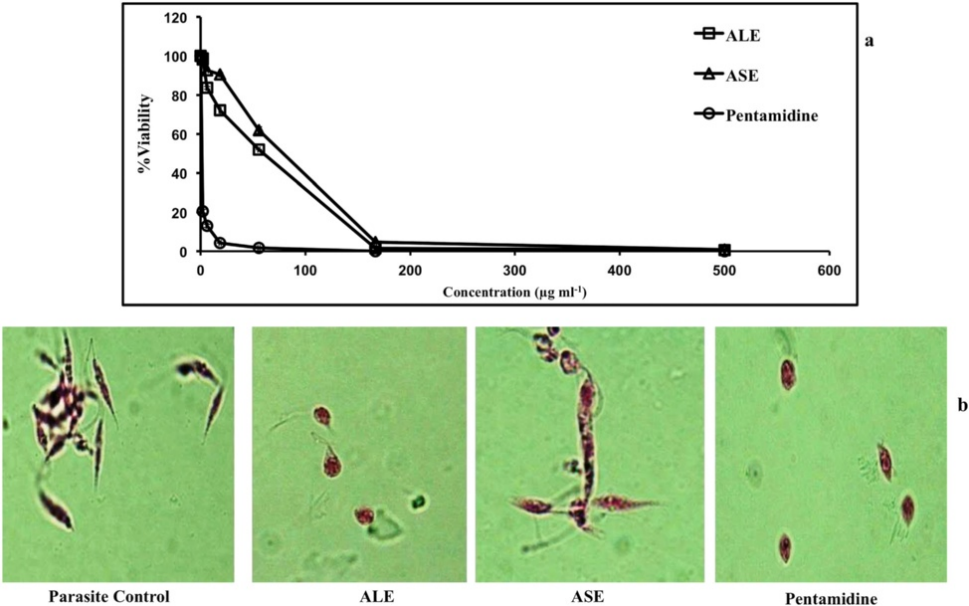
**Elaboration of anti-promastigote efficacy of** ***A. indica*** **fractions. (a)**. IC_50_ determination against *L. donovani* promastigotes. *L. donovani* promastigotes (2 × 10^6^ ml^−1^) were incubated with different concentrations of ALE, ASE and pentamidine as described in Methods. Each point corresponds to the mean ± SE of samples in triplicate and data is from one of three independent experiments. **(b)**. Light microscopy analysis of promastigote morphology. Promastigote forms of *L. donovani* were treated with ALE, ASE and pentamidine (500 μg ml^−1^) and stained with erythrosin B. Photomicrographs were recorded at 100 X under oil immersion.

IC_50_ was determined after incubation of *L. donovani* parasites with various concentrations of ALE, ASE or pentamidine. Treatment of *L. donovani* promastigotes with ALE and ASE resulted in a dose-dependent repression of parasite growth and IC_50_ was attained at 34 μg ml^−1^ and 77.66 μg ml^−1^, respectively while that of pentamidine was 1.4 μg ml^−1^ (Figure 2a and Table 1).

### ALE and ASE induce morphological alterations in *L. donovani* promastigotes

Morphological analysis of *L. donovani* promastigotes after treatment with the bioactive fractions revealed characteristic differences amongst the untreated and treated parasites. The untreated parasites retained their classical morphology being slender, elongated and flagellated (Figure 2b). In ALE treated promastigotes, changes pertaining to shape and size were observed. The cells became oval and exhibited an atypical appearance owing to substantial reduction in size, and shortening of flagella. ASE also induced similar changes in *L. donovani* promastigotes, though few cells retained their native morphology. Pentamidine treatment also resulted in a loss of prototypical morphology, with cells becoming oval and shrunken with degenerated flagella.

### PS externalization by ALE and ASE

**Figure 3:**
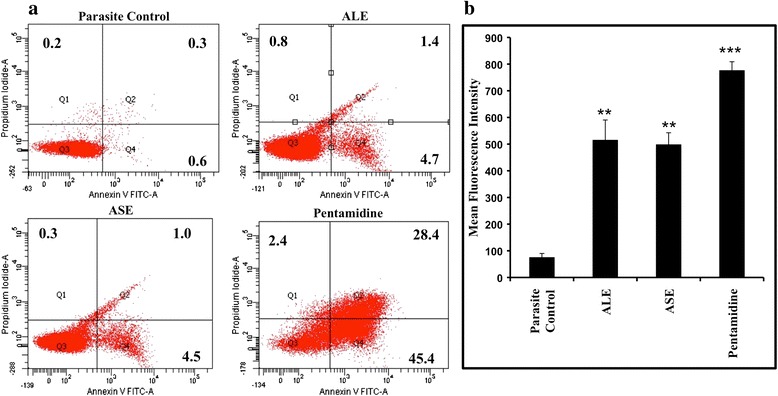
**Assessment of apoptosis inducing potential of bioactive fractions. (a)**. Determination of PS externalization in treated promastigotes. *L. donovani* promastigotes (2 × 10^6^ ml^−1^) were treated with 500 μg ml^−1^ of ALE, ASE and pentamidine for 72 h and dual-stained with Annexin-V-FLUOS and PI. The lower left quadrant represents live cells, upper left quadrant = PI positive, necrotic cells, lower right quadrant = Annexin-V-FLUOS positive, apoptotic cells, upper right quadrant = PI and Annexin positive, late apoptotic cells. The percentage of positive cells is shown in the respective quadrants. **(b)**. Probing of *in situ* DNA fragmentation by TUNEL assay. *L. donovani* promastigotes (2 × 10^6^ ml^−1^) were incubated in the absence or presence of ALE, ASE, and pentamidine (500 μg ml^−1^) for 72 h. The samples were processed according to instructions provided with ApoDirect kit (Roche), as described in Methods. ***P < 0.001 and **P < 0.01, in comparison to parasite control.

To ascertain the apoptotic or necrotic mechanism of induced cell death by ALE and ASE, bioactive fractions-treated promastigotes were co-stained with Annexin V-FLUOS and PI. Following treatment with ALE and ASE, 4.7% and 4.5% cells were found to be Annexin-V positive or apoptotic, respectively (lower right quadrant). 1.4% (ALE) and 1.0% (ASE) cells were found to be secondary apoptotic (the cells which first undergo apoptosis followed by disruption of cell membrane) as depicted in upper right quadrant. 0.8% (ALE) and 0.3% (ASE) cells were also observed to be PI positive (upper left quadrant). Thus, our results indicated that ALE and ASE exerted their antileishmanial activity primarily via apoptosis (Figure 3a). Pentamidine treatment resulted in extensive PS externalization with 45.4% cells being Annexin-V positive.

### ALE and ASE treatment cause DNA fragmentation

The DNA fragmentation, one of the hallmark traits of apoptosis, has been documented through TUNEL labeling, wherein the 3′OH groups are generated in abundance and serve as substrate for Tdt mediated fluorescence conjugated-dUTP binding. The histogram analysis revealed that whilst a small percentage of cells were TUNEL positive with mean fluorescence intensity (MFI) = 76 ± 14.0, in the promastigotes without any drug treatment, with and after pentamidine treatment, the MFI recorded was 777 ± 32.0 (*P* < 0.001). In ALE and ASE treated samples, the MFI increased to 516 ± 74.5 (*P* < 0.01) and 499 ± 43.66 (*P* < 0.01), respectively with respect to parasite control demonstrating apoptosis inducing capacity of the bioactive fractions (Figure 3b).

### ALE and ASE induce an amplification in sub G_0_-G_1_ population

**Figure 4:**
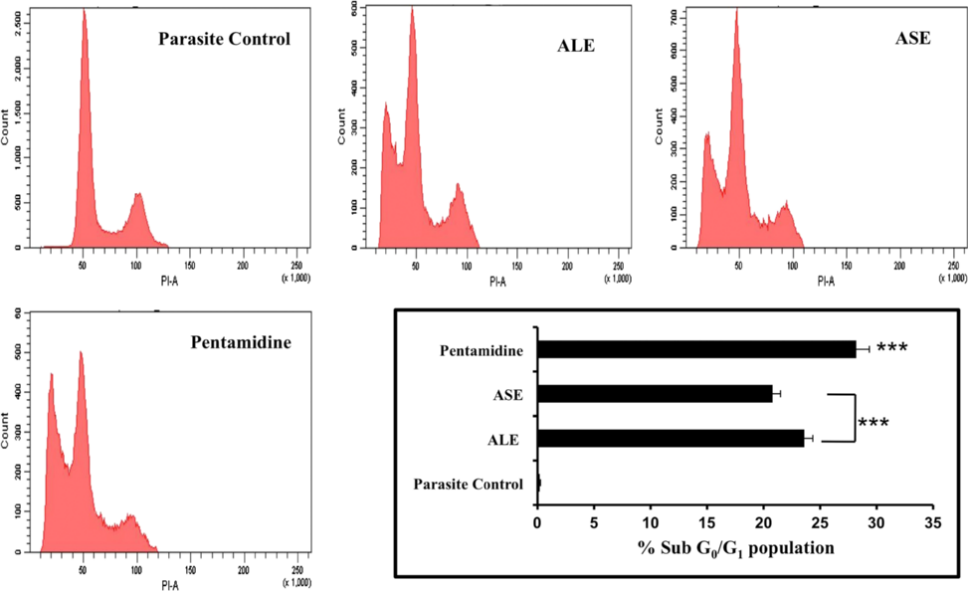
**Cell cycle analysis by PI staining.** *L. donovani* promastigotes (2 × 10^6^ ml^−1^) were incubated with ALE, ASE and pentamidine (500 μg ml^−1^). After 72 h, the samples were processed for cell cycle as described in Methods. The bar diagram depicts the percentage of cells in the sub G_0_-G_1_ phase. ***P < 0.001 with respect to parasite control.

Cell cycle analysis depicts the effect of different treatments on progression of cell cycle wherein presence of cells in sub G_0_-G_1_ phase indicates the occurrence of apoptosis. ALE and ASE triggered apoptosis in *L. donovani* promastigotes with an increase in sub G_0_-G_1_ population to 23.6% ± 0.75 (*P* < 0.001) and 20.8% ± 0.69 (*P* < 0.001), respectively in comparison to untreated parasite control where only 0.2% ± 0.05 cells were found to be apoptotic. Pentamidine treatment also resulted in an upsurge in percentage of apoptotic cells with 28.2% ± 1.15 (*P* < 0.001) cells in the sub G_0_-G_1_ phase (Figure 4).

### Bioactive fractions treatment depolarize mitochondrial membrane

**Figure 5:**
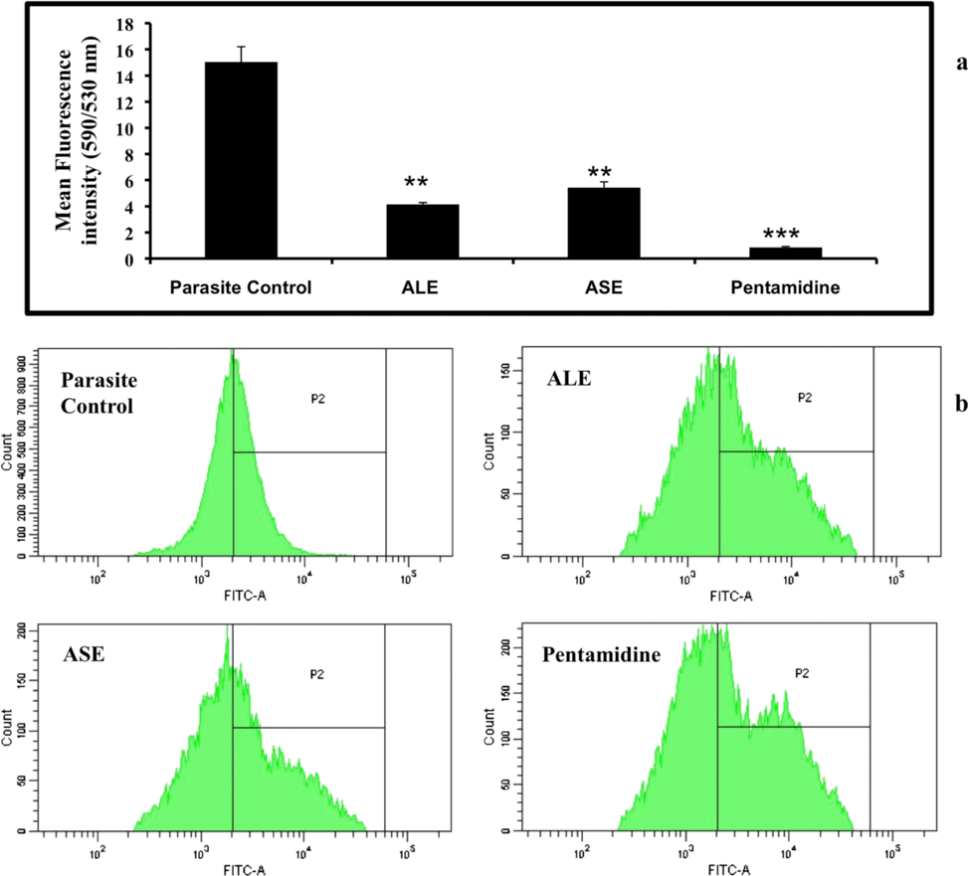
**Impact of ALE and ASE on cellular events leading to induction of apoptosis. (a)**. Effect of *A. indica* fractions on Ψm in *L. donovani* promastigotes. Untreated and treated parasites (2 × 10^6^ ml^−1^, 500 μg ml^−1^) were incubated for 72 h and stained with JC-1 and acquired in a flow cytometer. Red and green fluorescence positive cells were separately gated and their ratio was represented as relative Ψm values. ***P < 0.001, **P < 0.01 in comparison to parasite control. **(b)**. ROS generation in *L. donovani* promastigotes. *Leishmania* parasites (2 × 10^6^ ml^−1^) were grown in the presence or absence of bioactive fractions and pentamidine (500 μg ml^−1^) for 72 h and stained with H_2_DCFDA. Data was analyzed in the form of histograms where a shift in MFI in P2 gated region was recorded.

The disruption of mitochondrial membrane potential (Ψm) is an attribute of apoptosis. Since, hemoflagellate protozoans possess a single mitochondrion; loss of mitochondrial function becomes all the more critical for the survival of organism. To detect the changes in Ψm, JC-1 was used and the fluorescence intensity ratio obtained at 590 and 530 nm was determined as an indicator of transmembrane Ψm. In our study, ALE and ASE induced mitochondrial membrane depolarization as evidenced by fall in 590/530 nm ratio to 4.15 ± 0.16 (*P* < 0.01) and 5.41 ± 0.44 (*P* < 0.01), respectively versus untreated cells (15.03 ± 1.98). Pentamidine induced substantial depolarization by decreasing the 590/530 nm ratio to 0.84 ± 0.08 (Figure 5a).

### Treatment with bioactive fractions induce ROS generation

To examine the ROS generation post treatment with ALE and ASE in *L. donovani* promastigotes, H_2_DCFDA was used which fluoresce green upon cleavage with OH radicals and H_2_O_2_. The significant shift (*P* < 0.001 for all experimental groups) in mean fluorescence intensity (Figure 5b) in case of ALE (MFI = 10,037 ± 141.5) and ASE (MFI = 9935 ± 166) treated samples in comparison to untreated cells (MFI = 3481 ± 168) clearly depicted that ALE and ASE treatment successfully stimulated ROS production in *L. donovani* promastigotes. ROS production was also observed following treatment of *L. donovani* promastigotes with pentamidine (MFI = 10,656 ± 92).

### ALE and ASE reduce intra-macrophagic amastigote viability

**Figure 6:**
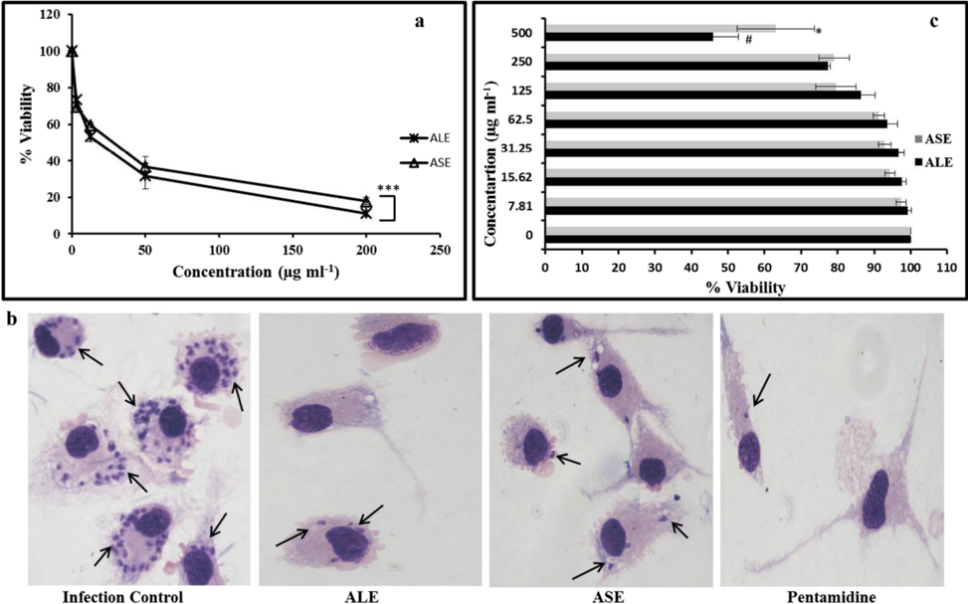
**Effect of ALE and ASE on intra-macrophagic amastigotes. (a)**. Estimation of anti-amastigote efficacy. Parasitized RAW 264.7 macrophages were cultured with ALE and ASE at different concentrations and percent reduction in amastigote growth was determined as described in Methods. ***P < 0.001in comparison to infected control. **(b)**. Microscopic imaging of *L. donovani* infected macrophages. Images depict giemsa stained intracellular amastigote forms residing in RAW macrophages. Arrow indicates the internalized parasites, where a decrease in amastigote number in treated samples is indicative of their anti-amastigote potential. **(c)**. Cytotoxicity of ALE and ASE against mammalian macrophages. RAW 264.7 cells (2 × 10^6^ ml^−1^) were incubated with ALE and ASE (0 to 500 μg ml^−1^) and cell viability was ascertained by MTT assay. Data shown are from one of the three independent experiments and are expressed as mean ± SEM of samples in triplicates. *P < 0.05, #*P* < 0.01 with respect to macrophage control without any treatment.

Upon entering the mammalian host, *Leishmania* parasites transform into the amastigote stage that reside inside the phagolysosomal vacuoles of macrophages. *Leishmania* amastigotes thus being the biologically and clinically relevant form, it was imperative to assess the anti-amastigote potential of our bioactive fractions. Accordingly, the anti-amastigote efficacy was assessed in RAW 264.7 macrophages parasitized by *L. donovani* promastigotes. There was a dose-dependent decrease in amastigote burden both with ALE and ASE treatment and IC_50_ (μg ml^−1^) was achieved at 17.66 ± 2.88 and 24.66 ± 4.98 μg ml^−1^, respectively. In case of pentamidine, IC_50_ was 0.87 ± 0.016 μg ml^−1^ (Figure 6a, Table 2). The reduction in amastigote burden was also visualized microscopically and was well evident in photomicrographs of giemsa stained untreated and treated macrophages (Figure 6b).

**Table 2:** **Bioactivity of ALE and ASE against** ***L. donovani*** **promastigotes and amastigotes as well as RAW 264.7 macrophages**

***A. indica*** **fractions/compounds**	**IC**_**50**_ **(μg ml** ^**−1**^ **) promastigotes**	**IC**_**50**_ **(μg ml** ^**−1**^ **) amastigotes**	**CC**_**50**_ **(μg ml** ^**−1**^ **) macrophages**	**SI**
**ALE**	34 ± 0.81	17.66 ± 2.88	461 ± 7.07	26.10
**ASE**	77.66 ± 3.13	24.66 ± 4.98	>500	-
**Pentamidine**	1.4 ± 0.249	0.87 ± 0.17	45.0 ± 2.80	51.72

Values represent Mean ± SE, SI = Selectivity Index (calculated as described in Methods), “-” indicates that SI could not be determined.

### Toxicity profile of the bioactive fractions and selectivity over host macrophages

Cytotoxic potential of the two bioactive fractions along with pentamidine was evaluated against RAW 264.7 macrophages to ensure their safety profile. The macrophages were incubated with the bioactive fractions at different concentrations (0–500 μg ml^−1^) and cell viability was assessed via MTT assay in which MTT is enzymatically reduced into formazan crystals which is subsequently dissolved in organic solvent(s) to generate pink to purple colour. The intensity of the colour produced is directly proportional to cell viability. With ALE, the CC_50_ obtained was 461 ± 7.07 and SI was 26.10. However, in case of ASE, no adverse cytotoxicity was observed and even at 500 μg ml^−1^, more than 50% cells were viable. In case of pentamidine, SI was found to be 51.72 (Figure 6c, Table 2).

### GC-MS analysis of ALE and ASE

Plant secondary metabolites present in ALE and ASE, which may have been responsible for the observed leishmanicidal effect, were analyzed by GC-MS analysis. The total compounds detected in ALE and ASE were 49 and 58, respectively out of which the major constituents are listed in Table 3. The complete GC-MS profile is elaborated in Additional files 1 and 2.

**Table 3:** **Major constituents of ALE and ASE as identified by GC-MS**

**S. no.**	**RT**	**%Area**	**Compound**
**ALE**			
1	39.212	25.21	1,2-Benzenedicarboxylic acid
2	47.709	16.95	Stigmasterol acetate
3	29.394	7.90	Palmitic acid
4	48.222	7.47	Ergosterol acetate
5	32.636	5.90	Cis,cis,cis-7,10,13-Hexadecatrienal
**ASE**			
1	32.938	12.96	(E)-9-Octadecenoic acid ethyl ester
2	49.151	10.45	19-Hydroxyandrost-5-en-3-yl acetate
3	9.353	8.56	1,2,3-Propanetriol
4	32.775	7.40	Linoleic acid
5	39.183	7.14	1,2-Benzenedicarboxylic acid

### Bioactive fractions reduce liver and spleen parasite burden

**Figure 7:**
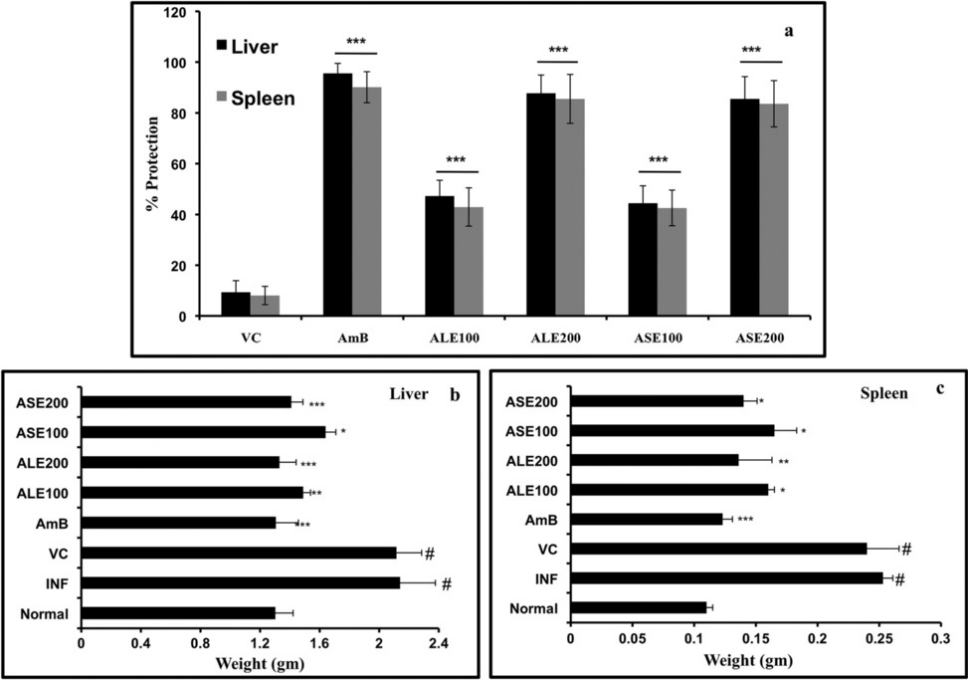
***In vivo*** **antileishmanial efficacy of** ***A. indica*** **fractions. (a)**. Determination of parasite burden. 10 weeks infected BALB/c mice were treated with ALE, ASE and AmB and were assessed for parasite burden 2 weeks post treatment as described in Methods. Graph depicts percent protection exhibited by the different treatment groups. ***P < 0.001 in relation to vehicle control for both liver and spleen experimental data. VC = Vehicle control, AmB = Amphotericin B, ALE100 = ALE at 100 mg/kg bw, ALE200 = ALE at 200 mg/ kg bw, ASE100 = ASE at 100 mg/kg bw, ASE200 = ASE at 200 mg/ kg bw. **(b)** Reduction in liver weight after treatment with bioactive fractions. 2 weeks post treatment, all the animals were sacrificed and intact livers were surgically isolated and weighed to assess the variation amongst the different experimental groups. #indicates significant (*P* < 0.001) enhancement in liver weight in INF and VC groups in comparison to normal mice. For all other groups ***P < 0.001, **P < 0.01, and *P < 0.05 in relation to infection control group. **(c)** Restoration of spleen weight after treatment with ALE and ASE. Spleen from all the animals were isolated and weighed aseptically to determine any changes following ALE and ASE treatment. # denotes *P* < 0.001 for INF and VC control group with respect to naïve mice. ***P < 0.001, **P < 0.01 and *P < 0.05 in comparison to INF group.

To assess the *in vivo* antileishmanial potential of ALE and ASE, 10 weeks-infected BALB/c mice were treated daily up to 2 weeks with ALE or ASE at 100 and 200 mg/kg bw. The mice were euthanized 2 weeks post-treatment. The antileishmanial efficacy was evaluated as fall in liver and spleen parasite burden and percent protection rendered by the different experimental groups was determined. As shown in Figure 7(a), mice that received only vehicle control (VC) exhibited the lowest levels of protection in liver as well as spleen, corresponding to 9.33% ± 4.58 and 8.03% ± 3.62, respectively. ALE conferred significant protection to infected mice (liver = 87.76% ± 7.19, *P* < 0.001, spleen = 85.55% ± 9.66, *P* < 0.001) at 200 mg/kg bw dose. At lower dose, moderate levels of protection were observed in the liver (47.19% ± 6.22) and spleen (42.93% ± 7.51) though the levels were statistically significant in comparison to VC (*P* < 0.001). At 200 mg/kg bw, ASE also exhibited significant (*P* < 0.001) and comparable protective efficacy with protection levels of 85.54% ± 8.8 and 83.62% ± 9.14 in liver and spleen, respectively. At lower dose, *i.e.*, 100 mg/kg bw, ASE endowed the lowest but significant protective efficacy (liver = 44.35% ± 6.88, *P* < 0.001, spleen = 42.56% ± 6.98, *P* < 0.001) of all the treatment groups. AmB, the known antileishmanial drug, induced maximum decrease in hepatic and splenic parasite burden with 95.61% ± 3.85 and 90.16% ± 6.14 protection, respectively.

The effectualness of AmB, ALE and ASE in curing *L. donovani* infection was also reflected in reduction of liver and spleen weights when compared to infection control (INF) group. As can be inferred from Figure 7(b and c), a significant increase in liver and spleen weight was well evident in INF and VC groups, indicative of heavy parasite burden in the organs in comparison to the normal mice. Following treatment with AmB, ALE and ASE, there was a considerable reduction in hepatic and splenic weight with respect to INF (*P* < 0.05). With ALE and ASE, reduction in liver and spleen weights was dose-dependent and treatment with higher doses was more effective in restoring the hepatic and splenic weights in normal range.

### ALE and ASE treatment in *L. donovani* infected BALB/c mice induces DTH response

**Figure 8:**
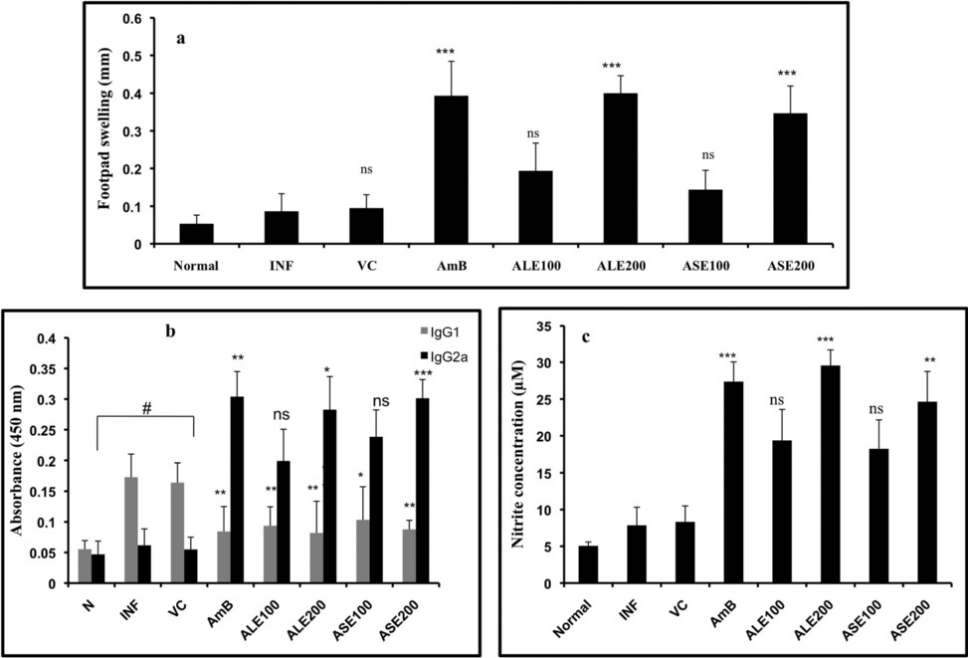
**Assessment of Th1 modulatory potential of ALE and ASE. (a)**. Evaluation of DTH response as an index of CMI. DTH responses were measured 2 weeks post treatment with the bioactive fractions and AmB. The response is expressed as difference (mm) between the thickness of right food pad (injected with 800 μg ml^−1^ of FT) and the left foot pad (injected with PBS), measured after 24 h. ***P < 0.001 with respect to INF, ns-not significant. **(b)**. Serum levels of IgG isotypes in bioactive fractions treated BALB/c mice. At 2 weeks post-treatment, the serum samples from different groups were assayed for *Leishmania* antigen specific IgG2a and IgG1 antibodies by ELISA. ***P < 0.001, **P < 0.01 and *P < 0.005 with respect to INF. # indicates statistical significance in INF and VC groups with respect to normal control in IgG1 plots. **(c)**. Estimation of NO generation *in vivo.* NO was quantified as nitrite by Griess reaction in supernatants of SLA-stimulated splenocytes derived from the indicated groups. ***P < 0.001, **P < 0.01 and ns-not significant with respect to infection control.

Cure of VL is generally concurrent with restoration of an effective cell-mediated immune response and the known chemotherapy also concomitantly establishes parasite clearance and host immune modulation towards Th1 type [4]. Thus, acquisition of DTH post successful treatment is a clear indication of Th1 polarization. Therefore, we evaluated whether the bioactive fractions promoted DTH responses in *L. donovani* infected BALB/c mice. Both ALE and ASE (200 mg/kg bw) exhibited pronounced DTH reactivity (*P* < 0.001) with ALE inducing the strongest DTH response, comparable to AmB (Figure 8a). No significant induction of DTH response was evidenced in ALE and ASE (100 mg/kg bw) as well as in VC group.

### Bioactive fractions treatment results in enhanced IgG2a levels and decline in the production of IgG1 antibodies

IgG2a (induced by INF-γ) and IgG1 (induced by IL-4) serve as surrogate markers for Th1 and Th2 activation, respectively. Sera from ASE (200 mg/kg bw) treated mice contained the highest levels IgG2a that was 4.9 fold higher than the INF (*P* < 0.001), closely followed by AmB (4.8 fold, *P* < 0.01) and ALE (4.6 fold, *P* < 0.05). The lower doses of bioactive fractions also stimulated the production of IgG2a. ASE and ALE (100 mg/kg bw) induced 3.9 and 3.2 fold, respectively more IgG2a antibody than infected mice, however the difference was not statistically significant. Antigen specific IgG1 levels were elevated in INF and VC groups in comparison to the untreated mice (*P* < 0.001). IgG1 level was substantially reduced in treated groups (*P* < 0.05) with little variation amongst the two doses (*P* < 0.05) (Figure 8b).

### Resolution of leishmaniasis by ALE and ASE is mediated by NO production

*Leishmania* impairs NO generation, which is the principle microbicidal molecule produced by macrophages in response to Th1 cytokines. Th1 polarization in ALE and ASE treatment correlated with increase in NO production. Minimal levels of NO were detected in naïve mice (5.07 μM). ALE (200 mg/kg bw) treatment led to maximum NO production, 29.57 μM versus 7.85 μM as detected in infection control group (*P* < 0.001). NO was also induced by ASE (24.64 μM, *P* < 0.01) at 200 mg/ kg bw. At 100 mg/kg bw, both ALE and ASE elevated nitrite levels to 19.38 μM and 18.23 μM, respectively which was non-significant in comparison to infection control. AmB treatment also resulted in upregulation of NO production to 27.38 μM (*P* < 0.001) (Figure 8c).

### ALE and ASE treatment induce lymphoproliferation in *L. donovani* infected BALB/c mice

**Figure 9:**
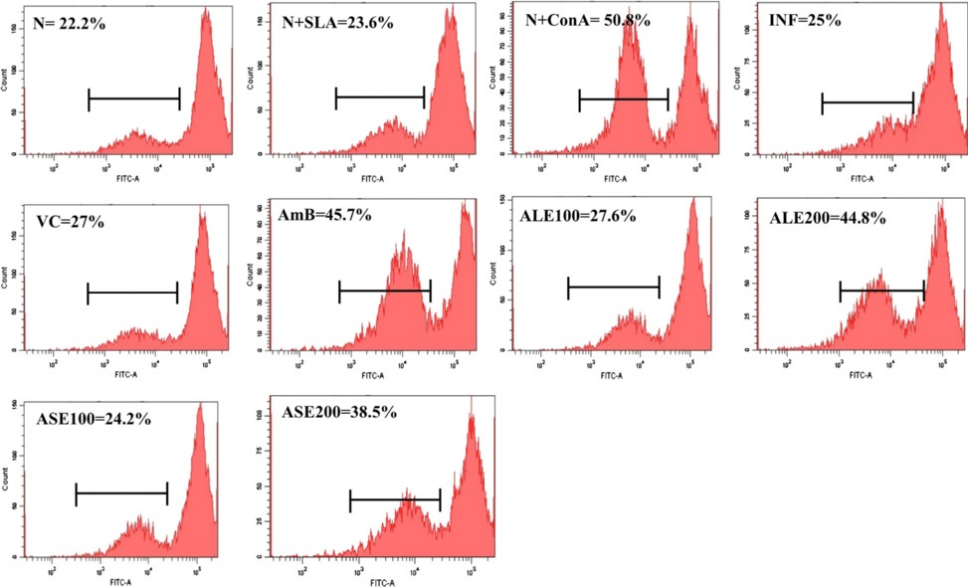
**Analysis of T-cell proliferation in splenocytes.** Splenocytes from respective experimental groups were re-stimulated with SLA and assessed for lymphoproliferation as described in Methods. Inset shows respective percentages of CFSE-positive cells.

During active VL, T cells are driven towards anergy to leishmanial antigens, denoting defective CMI. Thus, we were prompted to evaluate T cell proliferation responses in splenocytes of *L. donovani* infected mice. Proliferation of T cells after treatment with different groups was estimated by CFSE labeling of splenocytes. Since, CFSE intensity is reduced by half at each cell division; CFSE dilution assay provides an apparent measure of cell proliferation. Splenocytes from different experimental groups were *in vitro* re-stimulated with SLA and post 48 h of CFSE labeling, the cells were analyzed by flow cytometry. The percentage of normal cells (without SLA stimulation) that underwent division was 22.2% ± 1.34. SLA stimulated normal, infection control and VC groups exhibited 23.6% ± 0.28, 25.0% ± 0.31, and 27.0% ± 0.89 lymphoproliferation, respectively. Con A stimulated normal cells exhibited the highest lymphoproliferative response with 50.8% (±3.18) dividing cells. ALE (200 mg/kg bw) showed enhanced T cell proliferation (44.8% ± 1.90, *P* < 0.01) followed by ASE (38.5% ± 1.60, *P* < 0.01) upon *in vitro* restimulation with SLA. At 100 mg/ kg bw both ALE (27.6% ± 7.85) and ASE (24.2% ± 3.31) exhibited only partial lymphoproliferative response. Splenocytes from AmB treated mice also demonstrated stimulation of T cells with 45.7% (±0.93) cells undergoing division (Figure 9).

### ALE and ASE treatment induce increase in number of T lymphocytes

**Figure 10:**
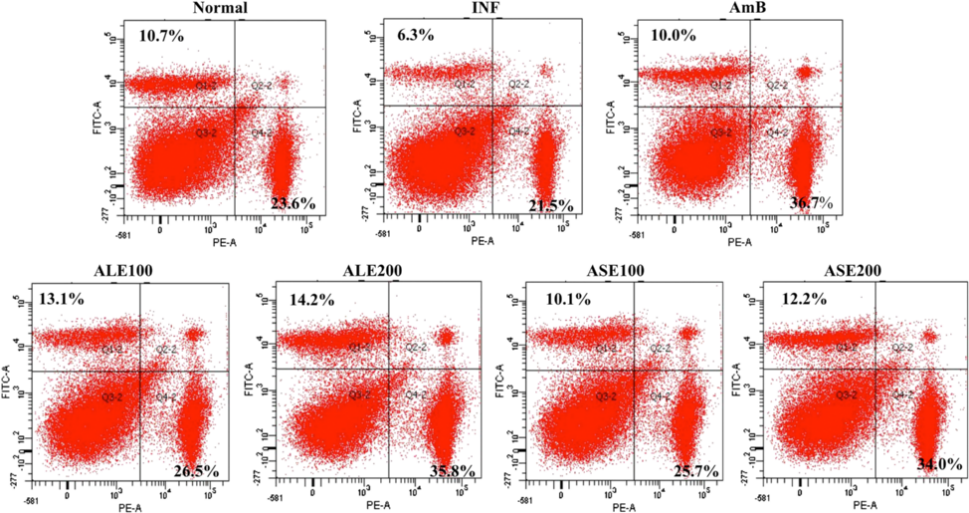
**CD4** ^**+**^ **and CD8** ^**+**^ **upregulation induced by bioactive fractions.** Splenic T lymphocytes from different treatment groups were co-stained with PE-conjugated antimouse-CD4 and FITC conjugated antimouse-CD8 antibodies. Results are expressed as dot plots and are representative of one of the two independent experiments performed. Inset shows percentages of CD4^+^ T cells (lower right) and CD8^+^ T cells (upper left) in respective quadrants.

As T cells contribute in resolving visceral infection, phenotypic analysis of CD4^+^ and CD8^+^ T lymphocytes of spleen was performed by flow cytometry. Spleen cells from different treatment groups were isolated, stained with antimouse-CD4-PE and antimouse-CD8-FITC, followed by acquisition in a flow cytometer along with appropriate controls. As deduced from quadrant statistics, the CD4^+^ and CD8^+^ T cells expressing individual populations were 23.6% ± 1.4 and 10.7% ± 0.8, respectively in naïve mice, which was down modulated in infected mice (CD4^+^ = 21.5 ± 1.2, CD8^+^ = 6.3% ± 1.5). All the treatment groups augmented CD4^+^ T cell population (Figure 10), with AmB resulting in maximum modulation (36.7% ± 1.10, *P* < 0.001). A comparable increase (35.8% ± 1.12, *P* < 0.001) was observed in ALE (200 mg/kg bw) treated mice. ASE treatment also induced pronounced elevation in CD4^+^ phenotype by increasing the population up to 34% ± 0.67 (*P* < 0.01). At lower dose of ALE and ASE, CD4^+^ percentage was restored to normalcy (ALE = 26.5% ± 1.85, ASE = 25.7% ± 0.76). AmB treatment reinstated the CD8^+^ population to normal level (10% ± 1.25). ALE (200 mg/kg bw) demonstrated an enhanced expression (14.2% ± 1.12, *P* < 0.01) whereas at 100 mg/kg bw, CD8^+^ population was found to be 13.1% ± 2.9 (*P* < 0.01). In case of ASE treatment, 100 mg/kg bw dose restored the CD8^+^ expression to normal range (10.1% ± 1.1) whereas 200 mg/kg bw dose upregulated the percentage of CD8^+^ population to 12.2% ± 1.6 (*P* < 0.05). Thus, the data indicates immunopotentiating effects of ALE and ASE.

### *A. indica* bioactive fractions enhance surface co-expression of CD80/CD86

**Figure 11:**
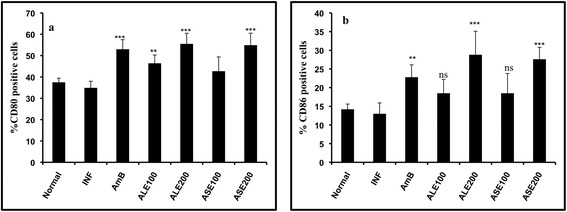
**Analysis of CD80 and CD86 expression on macrophages.** Peritoneal macrophages from naïve, infected and variously treated mice were dual-stained with antimouse-CD80-APC and antimouse-CD86-PeCy7. Bar graphs depict changes in **(a)** CD80 and **(b)** CD86 expression in the different groups. ***P < 0.001, **P < 0.01, *P < 0.05 and ns- not significant with respect to infection control.

Interaction between T cell receptor and MHC is strengthened via co-stimulatory molecules, CD80 (B7.1) and CD86 (B7.2). The percentage of macrophages expressing CD80 and CD86 was upregulated following treatment with the bioactive fractions. ALE (200 mg/kg bw) elevated CD80 and CD86 expression profile to 55.5% (*P* < 0.001) and 28.8% (*P* < 0.001), respectively in comparison with infection control (CD80 = 34.9%, CD86 = 13%). ASE at 200 mg/kg resulted in significant increase in expression of CD80 and CD86 to 54.9% (*P* < 0.001) and 27.6% (*P* < 0.001), respectively. Whereas, at 100 mg/kg bw, the CD80 expression was enhanced up to 46.4% (*P* < 0.01) and 42.7% (*P* < 0.05), respectively with ALE and ASE. Treatment at 100 mg/kg bw resulted in partial modulation of CD86 expression. CD86 was restored to normal levels with both ALE (18.7%) and ASE (18.5%) at 100 mg/kg bw and the changes were not significant in relation to infection control (*P* > 0.05). AmB treatment particularly enhanced the CD80 and CD86 expression up to 53% (*P* < 0.001) and 22.8% (*P* < 0.01), respectively with respect to infection control. In macrophages from naïve mice, the levels of CD80 and CD86 were 37.5% and 14.2%, respectively (Figure 11a and b).

### Bioactive fractions induced production of Th1 cytokines

**Figure 12:**
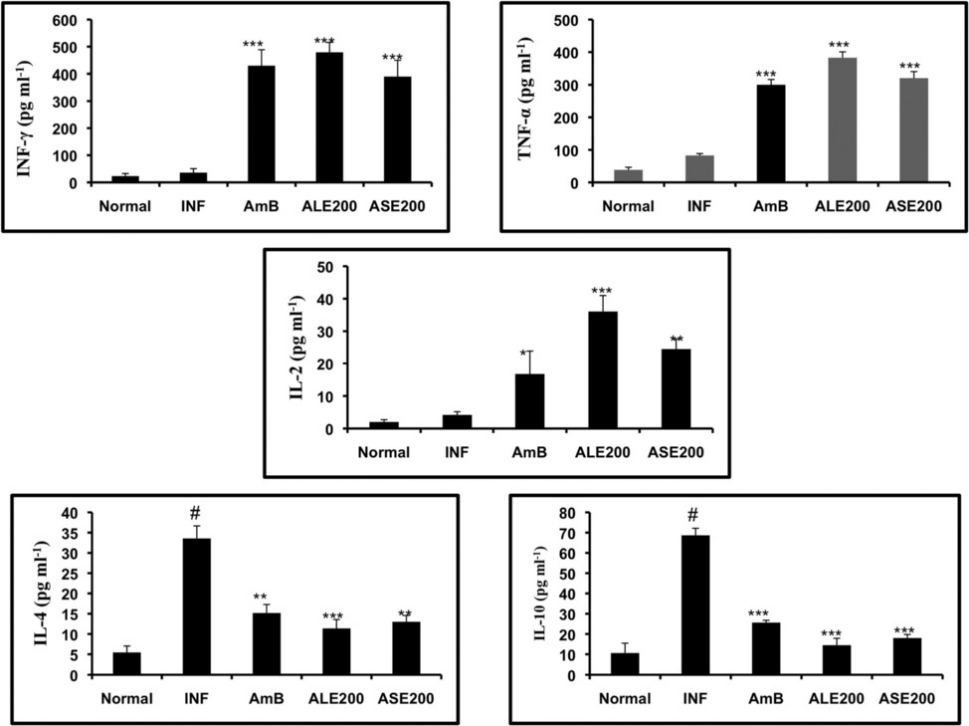
**Bioactive fractions altered cytokine expression profile in** ***L. donovani*** **infected mice.** Th1/Th2 cytokine levels were determined in SLA stimulated culture supernatants of spleen cells by cytokine bead array. ***P < 0.001, **P < 0.01 and *P < 0.005 with respect to infection control. # represents *P* < 0.001, statistical significance of INF with respect to normal group.

Th1/Th2 cytokine levels were quantified in culture supernatants of spleen cells from all the experimental groups. Animals treated with 200 mg/kg bw of ALE and ASE produced significant levels of INF-γ, TNF-α, and IL-2 with respect to infection control (*P* < 0.05). High concentrations of Th2 cytokines, IL-4 and IL-10 were observed in infection control group (*P* < 0.001) in comparison to naïve mice and a profound decline in IL-10 and IL-4 levels was witnessed in ALE and ASE treated groups (*P* < 0.01). AmB treatment also enhanced INF-γ, TNF-α, and IL-2 levels and declined secretion of IL-10 and IL-4 (Figure 12).

### Bioactive fractions were devoid of any toxic effects on liver and kidney

**Figure 13:**
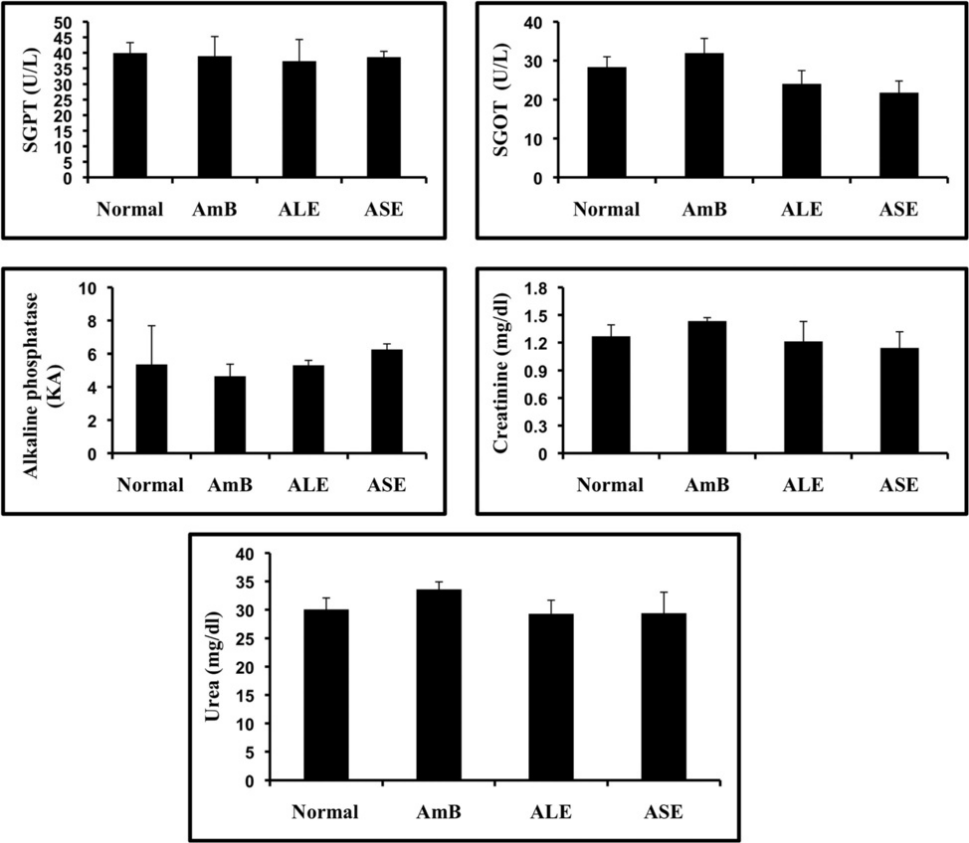
**Estimation of** ***in vivo*** **side effects of bioactive fractions.** ALE and ASE treated normal mice were bled and sera from the different experimental groups was used to estimate liver enzymes and kidney metabolites as described in Methods.

To assess the adverse toxicity of ALE and ASE, the fractions were administered in normal mice at 200 mg/kg bw and 2 weeks post treatment, sera from different groups were analyzed to assess the liver and kidney functions. No significant alterations in liver enzymes ALP, SGPT and SGOT were visible in ALE and ASE treated groups. Kidney function was also not impaired as indicated by normal range of serum urea and creatinine (Figure 13).

## Discussion

As proclaimed, plant derived secondary metabolites have become a focal point for discovery of antileishmanials with leishmanicidal and immunomodulatory properties. Previous studies which have divulged that leishmanicidal plants and plant secondary metabolites can potentiate key cellular immune responses, have proven to be coherent in the quest for more efficacious and less toxic antileishmanial drugs [4,27,28]. On guided lines, we evaluated the antileishmanial and immunostimulatory potential of *A. indica*. Major findings, from the present work undertaken were that ethanolic fractions of *A. indica* leaves (ALE) and seeds (ASE) inhibited the growth of *L. donovani* promastigotes via induction of apoptosis and provided protection to BALB/c mice against *L. donovani* infection via modulating the host immune responses without exerting any potential toxicity.

ALE and ASE repressed the growth of *L. donovani* promastigotes in a time- and dose-dependent manner, induced morphological alterations and their mode of induced cell death was cytocidal. *A. indica* extracts have been previously reported for their antileishmanial properties against *L. amazonensis* [10], *L. major* [8,9], and *L. donovani* clinical isolates and Dd8 strain [13]. We observed significant leishmanicidal activity of *A. indica* leaves and seeds ethanolic fractions on the *L. donovani* promastigotes (AG83), which have not been reported earlier. On the other hand, Sharma et al., [29] reported that methanolic extracts of *A. indica* leaves and stem bark were not active against *L. donovani* KE16. Such contrasting results may be accounted for differential virulence of the leishmanial strains (AG83 vs Dd8 vs KE16), difference in methods of extract preparation and also due to distinct profiles of secondary metabolites in these plant parts which varies in different geographic regions [30] including plants in the same country [31]. The leishmanicidal efficacy of *A. indica* fractions was found independent of quercetin, which has previously been reported to be antileishmanial ([32] and references therein). Quercetin was not included as a bioactive standard in any of the studies as it was not detected in the bioactive fractions by GC-MS analysis and this was also confirmed by thin layer chromatography (data not shown). However, isolation of quercetin [33] and of quercetin glycosides [34] has been done from aqueous extract of *A. indica* leaves. Thus, the absence of quercetin in ALE may be due to different extraction procedures. Other major constituents of ALE and ASE were identified by GC-MS analysis. The major constituent present in ALE was 1,2-Benzenedicarboxylic acid (25.21%) and was also detected in seed fraction (7.14%). This is a known component of *Ceratonia siliqua* essential oil, which exhibited antibacterial, antifungal and antitumor properties as well as of another medicinal plant, *Cleome burmanni* [35,36]. Stigmasterol acetate (16.95%), Ergosterol acetate (7.47%), stigmasterol (4.74%) along with γ-Sitosterol (1.3%) were detected in ALE. Both stigmasterol and γ-Sitosterol are endowed with anticancer, antimicrobial, antifungal, and antimycobacterial properties [37-40]. Stigmasterol and Ergosterol acetate are reported to be antileishmanial against *L. infantum* [41]. In ALE, palmitic acid (7.90%) was detected whereas linoleic acid (7.40%) and palmitic acid (6.84%) were identified in ASE. Both unsaturated [42] as well as saturated fatty acids have been shown to exhibit leishmanicidal activity and palmitic acid has been reported to be antileishmanial by Cunningham et al., [43]. Phytol (3.97%) and retinol (2.59%) were also detected in ALE. While, phytol presents a diverse spectrum of biological activities [44-46] and was also previously detected in antitrypanosomal extract of *Strychnos spinosa* [47] and antileishmanial extracts of *Arrabidaea chica* [48], Retinol (vitamin A alcohol) is endowed with antiplasmodial activity [49]. Another major component in ALE cis, cis, cis-7, 10, 13-Hexadecatrienal (5.90%) has been previously also detected in methanolic extract of *A. indica* leaves and was found to be larivicidal against *Aedes aegypti* [50]. Major components identified in ASE were not reported earlier for their biological activities, however, 9-Octadecenoic acid methyl ester (5.59%) and Ethylpalmitate (3.61%) have been related to antimicrobial properties [51,52].

*Leishmania* species are well known to exhibit apoptosis like changes in response to a diverse range of stimuli including plant extracts [16,18]. ALE and ASE treatment also triggered apoptosis like changes in *L. donovani* promastigotes characterized by PS externalization, induction of DNA fragmentation, cell cycle arrest at sub-G_0_/G_1_ phase, mitochondrial membrane depolarization and ROS generation. As discussed earlier, the anti-promastigote activity of *A. indica* is reported against different *Leishmania* species, but none of the studies have elucidated the mechanism of action of the bioactive extracts. *A. indica* extracts are known to stimulate apoptosis in many cancer cell lines [53,54] and in conformity, our results elucidate that *A. indica* bioactive fractions induce apoptosis mediated cell death in *Leishmania* promastigotes.

The anti-amastigote efficacy of the bioactive fractions was evaluated in a macrophage-amastigote model. The ALE and ASE reduced intra-macrophagic parasite growth of *L. donovani.* Aqueous extracts of *A. indica* have been demonstrated to potently inhibit axenic amastigote growth of *L. donovani* (Dd8 and other isolates) [13]. *A. indica* leaves ethanolic extract and dichloromethane fraction of nut tegument profoundly contained the growth of *L. amazonensis* amastigotes [10]. In accordance, our data also elaborated effectualness of *A. indica* bioactive fractions against *L. donovani* amastigotes. Selectivity index value was determined to assess cytotoxicity of the test fractions against mammalian macrophages. SI values above 10 were considered specific as described in case of other antiprotozoal compounds including those for *Leishmania* [55,56]. ALE possessed high selectivity as demonstrated by SI of 26.10 and ASE was not found to be toxic at all. This is in agreement with earlier studies where *A. indica* extracts were revealed to be marginally toxic against peritoneal macrophages [10] and human peripheral blood mononuclear cells [13].

ALE and ASE (200 mg/ kg bw) cured BALB/c mice of *L. donovani* infection with only marginal levels of parasites persisting in the liver and spleen along with restoration of liver and spleen weights to normal range. Partial resolution of infection was evident at lower doses of the bioactive fractions and changes in liver and spleen weight were less significant. *A. indica* methanolic extract successfully cured lesions in cutaneous leishmaniasis patients [11] and an aqueous extract of *A. indica* whole plant was demonstrated to reduce *L. donovani* Dd8 burden in BALB/c mice [12]. It is well established with VL that even a successful chemotherapy cannot completely eradicate all the parasites from infection sites; despite that patients with intact T cells display normal characteristics of clinical cure long after recovery [57,58]. Cure of infection in VL and presence of residual parasites has been divulged by others also [27,28]. The resolution of VL infection was accompanied by polarization of immune response towards Th1 type as evident from DTH, lymphoproliferative response, increased CD4^+^ and CD8^+^ T cell numbers and upregulation of serum IgG2a levels. Further, bioactive fractions enhanced NO production as well as elevated CD80/CD86 expression in macrophages. *A. indica* has been reported to possess immunomodulatory and adjuvant properties [59-61]. Our results were in accordance with these and study of different facets of CMI reinstated that potent immunomodulatory efficacy of *A. indica* fractions may be a major contributory factor in its leishmanicidal activity. AmB used as a positive control in this study, is known to be leishmanicidal by direct activity independent of host immune activation [62] as well as by exerting immune modulation where it has been demonstrated to induce DTH, enhance T cell proliferation, modulate serum IgG isotype levels, and induce NO production to skew the immune response towards Th1 type [23].

DTH response ultimately results in activation of macrophages via secretion of Th1 cytokines. In human VL, DTH also holds significance in the fact that perceptible clinical cure in the absence of a positive DTH response is often associated with relapse in infection [63]. Our data indicates that ALE and ASE (200 mg/kg bw) induced notable DTH response. Dominance of humoral response and depression of CMI is noteworthy during progressive VL. Serum levels of IgG2a and IgG1 indirectly affirm the activation of Th1 and Th2 cells, respectively. ALE and ASE (200 mg/kg bw) decreased the IgG1 levels and elevated IgG2a levels significantly which predicates the Th1 stimulating potential of the bioactive fractions. Further, the bioactive fractions treatment resulted in upregulation of NO production, which validated that parasite clearance was mediated via NO generation, the major microbicidal molecule responsible for intracellular killing of *Leishmania* parasites [27]. Restoration of an effective Th1 immune response was also investigated by assessing lymphoproliferation. Treatment with ALE and ASE annihilated T cell anergy evidenced in infected state, and a conspicuous T cell proliferation was apparent in all treated groups including AmB. Increase in T lymphocyte subpopulations (CD4^+^ and CD8^+^) after treatment with ALE and ASE also ascertained the immunomodulatory potency of the bioactive fractions. CD4^+^ T lymphocytes are already known to head VL recovery [64] but lately CD8^+^ T lymphocytes have been designated as equally important. During the course of VL infection, *L. donovani* suppresses the expansion and effector functions of CD8^+^ T cells [65]. CD8^+^ T cells have been shown to aid parasite clearance *in vivo* in BALB/c mice by direct lysis of infected cells as well as by recognizing *L. donovani* derived peptides from APCs [66]. Our bioactive fractions treatment led to the expansion of CD4^+^ T cells and restoration of CD8^+^ T cells to normal range.

CD80 and CD86 co-stimulatory molecules on APCs provide a vital link via interacting with CD28 on T cells that fortifies the interaction between T cell receptor and MHC. CD80-CD86 expression, which was muted during infection, was retrieved by treatment with ALE and ASE. Similar recovery of CD80-CD86 expression has been reported earlier in case of miltefosine [67] and pyrazinamide [68] treatment. In VL during disease progression, cytokine expression profile is skewed towards Th2 type, and successful restoration of immunity is reflected by induction of pro-inflammatory cytokines and polarization of immune response towards Th1 bias. The resolution of disease upon bioactive fractions treatment was accompanied by elevation in INF-γ, TNF-α and IL-2. INF-γ is potent macrophage activator, which promotes the class switching in B cells to produce IgG2a. Also, INF-γ in synergy with TNF-α, induces NOS II to produce NO the major microbicidal molecule against *Leishmania* and directs the immune response towards Th1 in the presence of IL-2 [69]. The induction of Th1 cytokines was accompanied by decline in levels of IL-4 and IL-10. ALE and ASE treatment reduced the levels of IL-4, which is responsible for production of IgG1 thus promoting Th2 phenotype. Our results also divulged elevated IgG2a serum levels in bioactive fractions treated group in comparison to IgG1, which can be well correlated to the enhanced IFN-γ production and down modulation of IL-4 secretion. Further, our *A. indica* bioactive fractions down regulated IL-10 production, a cytokine most closely related to disease exacerbation. Despite the ample production of IFN-γ in both murine and human VL, the infected hosts are usually handicapped in controlling the progressive disease, and the host inefficiency is attributed to high levels of IL-10 [4]. IL-10 impairs accessory cell functions including down-regulation of CD80 and CD86 expression leading to limited secretion of pro-inflammatory cytokines and suppression of NO production [70]. Our bioactive fractions up-regulated CD80-CD86 expression and NO production, which may be due to significant decline in IL-10 and elevation in INF-γ, and TNF-α level.

*In vivo* toxicity of the bioactive fractions was assessed at higher dose (200 mg/kg bw) in naïve mice. None of the treatment groups significantly altered serum SGOT, SGPT, ALP and urea as well as creatinine levels indicating that *A. indica* bioactive fractions were neither renal nor hepatotoxic. Non-toxic nature of *A. indica* has been illustrated in previous studies where the leaf preparations did not alter the serum ALP, SGOT and SGPT as well as urea and creatinine levels and no histological alterations were observed in liver and kidney sections of Swiss albino mice [71]. This was also supported by others [72,73] who reported that *A. indica* leaves and seeds were indeed hepato- and nephro-protective and their treatment did not alter liver and kidney functions.

Taken together, our data elaborates the antileishmanial efficacy of *A. indica* leaves and seeds ethanolic fractions, which is apparently potentiated by their immunostimulatory capacity.

## Conclusion

*A. indica* has been an integral part of Indian traditional medicine with each part of the plant endowed with miscellaneous medicinal properties. Here in, we demonstrated the potent leishmanicidal and immunomodulatory efficacy of *A. indica* leaves and seeds fractions, which advocates further standardization of these bioactive fractions for exploration as complementary medicine in the management of leishmaniasis either individually or more likely as a part of combination therapy.

## Additional files

Additional file 1:
**Constituents identified by GC-MS analysis of ALE.**
Additional file 2:
**Constituents identified by GC-MS analysis of ASE.**

## Abbreviations

VL: Visceral Leishmaniasis
ALE: *Azadirachta indica* leaves ethanolic fraction
ASE: *Azadirachta indica* seeds ethanolic fraction
GC-MS: Gas chromatography–mass spectrometry
CMI: Cell-mediated immunity
RPMI: Roswell Park Memorial Institute
PI: propidium iodide
FBS: Fetal bovine serum
DMSO: Dimethylsulfoxide
HEPES: 4-(2-hydroxyethyl)-1-piperazine-ethanesulfonic acid
LDU: Leishman donovan units
JHAEC: Jamia Hamdard Animal Ethics Committee
CPCSEA: Committee for the purpose of control and supervision of experiments on animals
PS: Phosphatidylserine
TUNEL: TdT (terminal deoxynucleotidyl transferase) mediated dUTP (deoxyuridine triphosphate) nick end labeling
OH: Hydroxyl
PCD: Programmed cell death
PBS: Phosphate buffered saline
NO: Nitirc oxide
Ψm: Membrane potential
JC-1: 5,59,6,69-tetrachloro-1,19,3,39-tetraethylbenzimidazolylcarbo-cyanineiodide
H_2_DCFDA: 2′, 7′- dichlorodihydrofluorescein-diacetate
ROS: Reactive oxygen species
DCF: 2′,7′-dichlorofluorescein
MFI: Mean fluorescence intensity
MTT: 3-(4,5-dimethylthiazol-2-yl)-2,5-diphenyl tetrazolium bromide
CC_50_: 50% cytotoxic concentration
SI: Selectivity index
IC_50_: 50% inhibitory concentration
FT: Freeze thawed antigen
SLA: Soluble leishmanial antigen
IgG: Immunoglubulin G
Th1: T helper type 1
DTH: Delayed type hypersensitivity
Con A: Concanavalin A
AmB: Amphotericin B
CFSE: Carboxyfluorescein succinimidyl ester
PE: Phycoerythrin
FITC: Fluorescein isothiocyanate
APC: Allophycocyanin
NOS II: Nitric oxide synthase II
APC: Antigen presenting cells
MHC: Major histocompatibility complex
SGPT: Serum glutamate pyruvate transaminase
SGOT: Serum oxaloacetate transaminase
ALP: Alkaline phosphatase
SEM: Standard error of mean
RBC: Red blood cells
ANOVA: One-way analysis of variance
ABH: *A. indica* bark hexane fraction
