# Erratum to: Apoptosis mediated leishmanicidal activity of *Azadirachta indica* bioactive fractions is accompanied by Th1 immunostimulatory potential and therapeutic cure *in vivo*

**DOI:** 10.1186/s13071-016-1593-3

**Published:** 2016-05-27

**Authors:** Garima Chouhan, Mohammad Islamuddin, Muzamil Y. Want, Malik Z. Abdin, Hani A. Ozbak, Hassan A. Hemeg, Dinkar Sahal, Farhat Afrin

**Affiliations:** Department of Biotechnology, Parasite Immunology Laboratory, Faculty of Science, Jamia Hamdard (Hamdard University), New Delhi, 110062 India; Department of Biotechnology, Centre for Transgenic Plant Development, Faculty of Science, Jamia Hamdard (Hamdard University), New Delhi, 110062 India; Department of Medical Laboratories Technology, Faculty of Applied Medical Sciences, Taibah University, P.O. Box: 344, Universities Road, Medina, 30001 Saudi Arabia; Malaria Research Group, International Centre For Genetic Engineering and Biotechnology, Aruna Asaf Ali Marg, New Delhi, 110067 India

## Erratum

Unfortunately, the original version of this article [1] contained an error within the author list. Affiliation 1 was incorrectly assigned to Dr Farhat Afrin. The correct author list and affiliations can be found here. In addition the acknowledgment of the original article is missing supporting information. The correct acknowledgements can be found below.
