# Supplementary material for: Apoptosis mediated leishmanicidal activity of Azadirachta indica bioactive fractions is accompanied by Th1 immunostimulatory potential and therapeutic cure in vivo
Source: Parasit Vectors. 2015 Mar 26;8:183. doi: 10.1186/s13071-015-0788-3 (PMC4381458; doi:10.1186/s13071-015-0788-3)
Supplement: Additional file 1: — Constituents identified by GC-MS analysis of ALE. [file 13071_2015_788_MOESM1_ESM.pdf]

Additional File 1. GC-MS constituents of ALE

S. No.	RT	%Area	Compound
1.	7.262	0.88	2-Pyrrolidinone
2.	16.444	0.32	Dodecanol
3.	17.177	0.07	Trans-β-Caryophyllene
4.	19.579	0.22	2,4-Ditert-butylphenol
5.	21.278	0.14	Pentadecene
6.	25.280	0.37	Myristic acid
7.	25.544	0.22	(-)-Loliolide
8.	26.612	1.04	Neophytadiene
9.	27.119	0.24	(2E)-3,7,11,15,-Tetramethyl-2-hexadecen-1-ol
10.	27.746	0.42	2-Hexadecen-1-ol
11.	**29.394**	**7.90**	**Palmitic acid**
12.	29.637	0.31	α-Octadecene
13.	31.140	0.26	d-Galactose, dioctyl mercaptal
14.	31.930	3.97	Phytol
15.	**32.636**	**5.90**	**cis,cis,cis-7,10,13-Hexadecatrienal**
16.	32.957	0.84	Stearic acid
17.	33.278	0.2	Heneicosanol
18.	**39.212**	**25.21**	**1,2-Benzenedicarboxylic acid**
19.	42.299	0.4	1,2-Benzenedicarboxylic acid didecyl ester
20.	42.759	0.74	1,2-Benzenedicarboxylic acid diisodecyl ester
21.	43.147	0.32	Pregn-4-ene-3,20-dione
22.	43.608	0.10	Actetamidocyclohexane
23.	43.808	0.04	2-(3,7-Dimethyl-octa-2,6-dienyl)-4-methoxy phenol
24.	44.144	0.21	Heneicosane
25.	44.942	0.35	Tetratriacontane
26.	45.123	0.17	2,2,6,6-Tetramethyl-4-piperidinyl benzoate
27.	45.356	0.88	α-Tocopherol
28.	46.029	0.08	Zymosterol
29.	46.074	0.96	Retinol acetate
30.	46.398	0.84	Stigmasta-5,22-dien-3-ol
31.	46.854	1.30	γ-Sitosterol
32.	47.108	0.15	Retinoic acid
33.	47.256	2.33	Oxatricyclo[20.80.0(7,16)]triaconta-1(22),7(16),9,13,23,29-hexane
34.	**47.709**	**16.95**	**Stigmasterol acetate**
35.	48.019	4.74	Stigmasterol
36.	**48.222**	**7.47**	**Ergosterol acetate**
37.	48.586	0.28	2,6,10-Trimethyl,14-ethylene-14-pentadecne
38.	48.808	0.13	Secodammar-4(28)-en-3-oic acid
39.	48.951	0.76	Humulene-1,6-dien-3-ol

40.	49.391	2.59	Retinol
41.	49.814	0.51	Medroxyprogesterone acetate
42.	50.133	0.30	Stigmasta-5,22-dien-3-β-ol
43.	50.394	0.45	Strophanthidol
44.	50.755	0.25	Acetoxypregnenolone acetate
45.	51.142	0.4	Trans retinol-acetate
46.	51.325	1.22	Hydroxycholestrol
47.	51.913	0.36	6-(1,5-Dimethyl-hex-4-enyl)-1,6-dihydroxy-1, 8a –dimethyl-3-oxo-1,2,3, 3a,5a,6.7,8,8a,9,10,10a-dodecahydrodicyclopenta[a,e]cyclooctene
48.	52.425	0.39	N 2-(3-hydroxy-4,4,10,13,14,-pentamethyl-2,3,4,5,6,7,10,11,12,13,14,15,16,17-tetradecahydro-1H-cyclopenta[a]phenanthryl-methanesulfonic acid
49.	53.373	5.80	Butenoic acid,2-methyl,1a-2,4,4a,5,9,-hexahydro-4,4a,6-trimethyl-3H-oxiremo[8,8a]napthalo[2,3-b]furan-5-yl ester

Major constituents are highlighted in bold
