# Supplementary material for: Apoptosis mediated leishmanicidal activity of Azadirachta indica bioactive fractions is accompanied by Th1 immunostimulatory potential and therapeutic cure in vivo
Source: Parasit Vectors. 2015 Mar 26;8:183. doi: 10.1186/s13071-015-0788-3 (PMC4381458; doi:10.1186/s13071-015-0788-3)
Supplement: Additional file 2: — Constituents identified by GC-MS analysis of ASE. [file 13071_2015_788_MOESM2_ESM.pdf]

Additional File 2. GC-MS constituents of ASE

S.No.	RT	%Area	Compound
1.	6.066	0.18	Decane
**2.**	**9.353**	**8.56**	**1,2,3-Propanetriol**
3.	11.127	0.06	Undecanol
4.	11.342	0.09	Dodecane
5.	16.427	0.17	3-Hexadecene
6.	19.568	0.27	2,4-Ditert-Butylphenol
7.	21.278	0.31	E-14-Hexadecenal
8.	25.296	0.18	Tetradecanoic acid
9.	25.654	0.28	Octadecene
10.	26.607	0.08	2,6,10-Trimethyl,14-ethylene-14-pentadecne
11.	28.393	2.04	n-Hexadecanoic acid methyl ester
**12.**	**29.542**	**6.84**	**Palmitic acid**
13.	29.726	3.61	Ethyl palmitate
14.	31.705	5.59	Octadecenoic acid, methyl ester
15.	32.152	1.31	Stearic acid methyl ester
**16.**	**32.775**	**7.40**	**Linoleic acid**
**17.**	**32.938**	**12.96**	**(E)-9-Octadecenoic acid ethyl ester**
18.	33.219	0.89	Octadecanoic acid
19.	33.372	2.12	Ethyloctadecanoate
20.	36.688	0.5	Stearic acid ethyl ester
21.	37.726	0.09	Fumaric acid, 2-dimethylaminoethylnonyl ester
22.	39.183	7.14	1,2-Benzenedicarboxylic acid
23.	39.778	0.28	Hexadecanoinc acid ethyl ester
24.	40.188	0.13	2-Butoxyethyloleate
25.	40.958	0.08	Kauren-19-yl-acetate
26.	41.162	0.43	Tetratriacontane
27.	41.992	0.59	3-Hydroxy-6-isppropenyl-4,8a-dimethyl-1,2,3,4,5,6,7,8,8a-octahydro-2-napthalenyl acetate
28.	42.297	0.42	Ethyl docosanoate
29.	42.632	0.64	Squalene
30.	43.280	0.66	Hexatriacontane
31.	43.600	0.16	Linolenic acid methyl ester
32.	44.613	0.22	12-Hydroxy-16,17-dimethyl-pregn-4-ene
33.	44.942	0.57	Tetratetracontane
34.	45.058	0.01	Docosanol
35.	45.187	0.34	4,4-7a-Trimethyl-1,3-dimethylene
36.	45.365	0.3	δ-Corlin
37.	45.945	0.14	(3E,5E,7E)-6-Methyl-8-(2,6,6-trimethyl-1-cyclohexen-1-yl)-3,5,7-octatrien-2-one
38.	46.108	3.87	6β-Hydroxymethandienone
39.	46.440	0.51	Retinol acetate
40.	46.740	0.6	(E,E,E)-3,7,11,15-Tetramethylhexadeca-1,3,6,10,14-pentane

41.	46.861	0.68	γ-Sitosterol
42.	47.237	0.5	Oxatricyclo[20.80.0(7,16)]triaconta-1(22),7(16),9,13,23,29-hexane
43.	47.592	0.46	Ergosta-7,22-dien-3-β-ol, acetate
44.	47.913	1.29	Methyl-3-acetoxy-12-keto-18-norcholanate
45.	48.111	1.63	2,4,6-Triisopropylbenzoylchloride
46.	48.558	2.53	Androst-5-ene-3,19-diol acetate
**47.**	**49.151**	**10.45**	**19-Hydroxyandrost-5-en-3-yl acetate**
48.	49.306	2.01	Santolina expoxide
49.	49.760	0.56	Menthofuran
50.	50.423	0.30	Strophanthidol-3,19-diacetate
51.	50.782	0.22	21-Acetoxy-3-pregenolone acetate
52.	51.053	0.61	Desoxycorticosterone acetate
53.	51.364	0.33	26-Hydroxycholesterol
54.	52.133	2.78	Acetoxy-bisnor-5-cholenic acid
55.	52.609	0.15	20-Ethynyl-5-pregen-3,20-diol
56.	53.227	1.56	Stigmasterol
57.	53.983	2.22	Butenoic acid,2-methyl,1a-2,4,4a,5,9,-hexahydro-4,4a,6-trimethyl-3H-oxiremo[8,8a]napthalo[2,3-b]furan-5-yl ester
58.	58.836	1.07	9-Octadecenoic acid (Z)-,2-hydroxyl-1,3-propanediyl ester

Major components are highlighted in bold
